# Berberine increases stromal production of Wnt molecules and activates Lgr5^+^ stem cells to promote epithelial restitution in experimental colitis

**DOI:** 10.1186/s12915-022-01492-z

**Published:** 2022-12-17

**Authors:** Zecheng Luo, Zihao Li, Zheng Liang, Lin Wang, Guanlin He, Dongdi Wang, Lei Shen, Zhengting Wang, Xiuying Ma, Funeng Geng, Haozhong Wang, Wenping Liu, Huijuan Liu, Baojie Li

**Affiliations:** 1 Institute of Traditional Chinese Medicine and Stem Cell Research, College of Basic Medical Sciences, Chengdu University of Traditional Chinese Medicine, Chengdu, China; 2 Bio-X-Renji Hospital Research Center, Renji Hospital, School of Medicine, Shanghai Jiao Tong University, Shanghai, China; 3 Shanghai Institute of Immunology, Department of Immunology and Microbiology, Shanghai Jiao Tong University School of Medicine, Shanghai, China; 4 Department of Gastroenterology, Ruijin Hospital, School of Medicine, Shanghai Jiao Tong University, Shanghai, China; 5 Good Doctor Pharmaceutical Group of Sichuan, Chengdu, 610000 Sichuan China; 6 College of Basic Medical Sciences, Chengdu University of Traditional Chinese Medicine, Chengdu, 610075 China

**Keywords:** Berberine, Colitis, ISC, Wnt, Circadian rhythm, Stromal cell

## Abstract

**Background:**

Inflammatory bowel diseases (IBDs) are characterized by sustained inflammation and/or ulcers along the lower digestive tract, and have complications such as colorectal cancer and inflammation in other organs. The current treatments for IBDs, which affect 0.3% of the global population, mainly target immune cells and inflammatory cytokines with a success rate of less than 40%.

**Results:**

Here we show that berberine, a natural plant product, is more effective than the frontline drug sulfasalazine in treating DSS (dextran sulfate sodium)-induced colitis in mice, and that berberine not only suppresses macrophage and granulocyte activation but also promotes epithelial restitution by activating Lgr5^+^ intestinal stem cells (ISCs). Mechanistically, berberine increases the expression of Wnt genes in resident mesenchymal stromal cells, an ISC niche, and inhibiting Wnt secretion diminishes the therapeutic effects of berberine. We further show that berberine controls the expression of many circadian rhythm genes in stromal cells, which in turn regulate the expression of Wnt molecules.

**Conclusions:**

Our findings suggest that berberine acts on the resident stromal cells and ISCs to promote epithelial repair in experimental colitis and that Wnt-β-Catenin signaling may be a potential target for colitis treatment.

**Supplementary Information:**

The online version contains supplementary material available at 10.1186/s12915-022-01492-z.

## Background

Inflammatory bowel diseases including Crohn’s disease and ulcerative colitis affect 0.3% of the global population and the incidence is on the rise [1–3]. The pathogenesis of IBD is complex and remains poorly understood. It is generally believed that disruption of the mucus barrier, change in microbiota, and sustained immune response, under the influence of the genetic makeup and the environments, are major etiological factors [2]. IBD causes diarrhea, rectal bleeding, abdominal pain, and weight loss and has complications such as colorectal cancer, eye and joint inflammation, and malnutrition [4]. The integrity of colonic mucosa is maintained by Lgr5^+^ intestinal stem cells (ISCs) located at the base of the crypts under homeostasis or repairing conditions [5], which are controlled by signaling molecules such as Wnt and BMP provided by the niche cells [6]. Mesenchymal stromal cells (referred to as stromal cells thereafter) constitute the major ISC niche in the colon [7].

Recent studies have shown that IBD pathogenesis may involve the circadian rhythms, which have been long known to control the activities of the digestive tract [8–10], including nutrient absorption, epithelial permeability, and motility [11–16]. The hypothalamus suprachiasmatic nucleus is the central circadian pacemaker that entrains to the light/dark cycle, feeding, and temperature [12, 17]. Other organs also display circadian rhythms and the central circadian clock controls the peripheral clocks via hormones such as melatonin and glucocorticoid [18]. At the molecular level, the circadian Clock-Bmal1 complex controls the expression of rhythm-related genes, which itself is tightly controlled by the Cry1/2 and Per2/3 feedback loops, as well as by the ROR and REV-ERB loops [19]. Moreover, it has been reported that shift work or polymorphism of *Per3* increases the risk of IBD [20, 21]. In addition, young untreated IBD patients show dysregulated expression of circadian genes including *Arntl* (encoding Bmal1) and *Clock* [22]. Recent studies have shown that disruption of circadian rhythms in macrophages has an impact on IBD development [23, 24]. However, how circadian rhythms contribute to IBD pathogenesis remains incompletely understood.

The ideal endpoint of IBD treatment includes immune resolution and epithelial restoration [25]. The available treatments mainly target immune cells and inflammatory cytokines, with a success rate of less than 40% [2, 26, 27]. Previous studies have explored the possibility of targeting epithelial cells to treat IBD, including the use of EGF and activation of TGFβ signaling to promote epithelial proliferation [28, 29]. However, such studies have been complicated by the oncogenic activity and/or pro-inflammatory activity of the treatments [2].

Emerging studies have shown that berberine (BBR) is effective in treating experimental colitis [30–33]. Berberine is an alkaloid and an active component of Chinese herbs used to treat gastroenteritis and bacterial dysentery [34–37], and is also being tested in clinical trials in treating IBD. BBR has been reported to suppress gut microbiota [32, 38, 39], improve barrier function [40, 41], and suppress T cells via AMPK [42], mTORC1 [43], NF-κB [44], and phospholipase A2 [45], and macrophages [46, 47]. In addition, BBR shows potentials in treating diabetes, high cholesterol, Parkinson disease, and cancer [48–52]. BBR may modulate lipid and glucose metabolism via activating AMPK [53–56].

Here, we compared BBR and sulfasalazine (SASP), a frontline anti-inflammation drug used to treat ulcerative colitis, and found that BBR was more effective in treating DSS-induced colitis in mice. Sulfasalazine is a prodrug that is cleaved by bacteria in the colon to liberate active compound 5-aminosalicylic acid, which suppresses inflammation via NF-κB signaling [57]. We showed that colitis pathogenesis involved decreased expression of genes controlling mineral absorption, metabolism, and circadian rhythms in addition to inflammation. The superior effect of BBR over SASP is likely attributable to BBR’s effects on ISC proliferation, evidenced by genetic tracing experiments, besides inflammation suppression. In vivo and cell culture studies show that BBR up-regulates the expression of several Wnt molecules in resident stromal cells, which are the major cellular component of the ISC niche, via circadian rhythms, to activate ISCs. These findings suggest that the stromal niche/ISC duo is a feasible target for colitis treatment and that the dual effects of BBR in suppressing inflammation and promoting epithelial restitution make it a strong candidate for the treatment of colitis patients.

## Results

### Berberine showed greater efficacy in colitis treatment than sulfasalazine

Several studies have shown that BBR is effective in treating colitis in mouse models. To understand how BBR executes its therapeutic effect, we first compared BBR with SASP. We determined the optimal doses of BBR and SASP in treating DSS-induced colitis in mice by using 50, 100, 150, or 200 mg/kg BBR or SASP to treat adult male mice (via gavage administration) at day 1 after DSS treatment. We found that BBR showed maximal therapeutic effect at 100 mg/kg while SASP at 200 mg/kg, judged by the changes in body weight, colon length, histological changes, the number of goblet cells, and the colitis score (Fig. S1A–H). However, BBR showed a lesser effect at 150 or 200 mg/kg (Fig. S1A–D), suggesting that it might have a toxic effect at high doses.

**Fig. 1:**
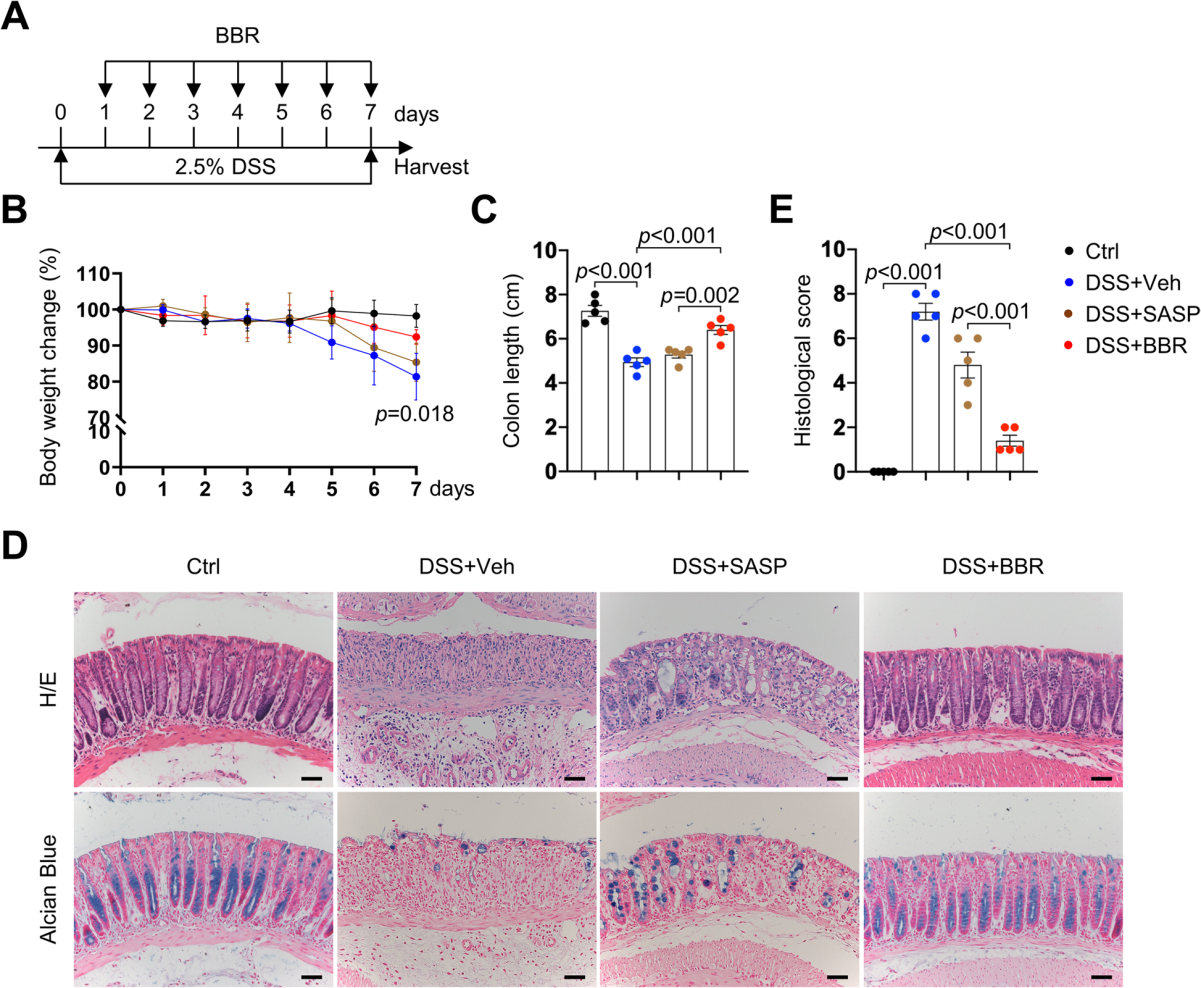
Comparison of BBR and sulfasalazine in treating colitis model mice. **A** Diagram showing the times for DSS and BBR treatment and colorectal sample collection. Colorectal samples were harvested after 7 days of DSS treatment in adult male mice, which were treated with BBR (100 mg/kg) or SASP (200 mg/kg) starting from day 1. **B**–**E** The body weights (**B**), colon length (**C**), and histological scores (**E**) were determined and H/E and Alcian Blue staining of rectal sections were performed (**D**). *n*=5 per group. Scale bars, 50 μm. Data are presented as means±SEM in **B**, **C**, and **E**. Unpaired two-tailed Student’s *t* test was applied in **B**, **C**, and **E**. *p*<0.05 was considered as statistically significant

We then compared the therapeutic effects of BBR (100 mg/kg) and SASP (200 mg/kg) head to head. We administrated BBR or SASP at day 1 or 7 after DSS treatment (Fig. 1A and Fig. S2A–E) and found that both BBR and SASP alleviated colitis phenotypes with BBR showing greater effects, under both conditions (Fig. 1B–E and Fig. S2A–E). We also compared BBR from two suppliers and found that they had similar effects on experimental colitis (Fig. S2F). Given that BBR has a similar molecular weight as SASP (372.822 vs 398.39), these results suggest that BBR is more potent than SASP in treating experimental colitis.

### Transcriptome analysis revealed new pathogenic aspects of colitis

**Fig. 2:**
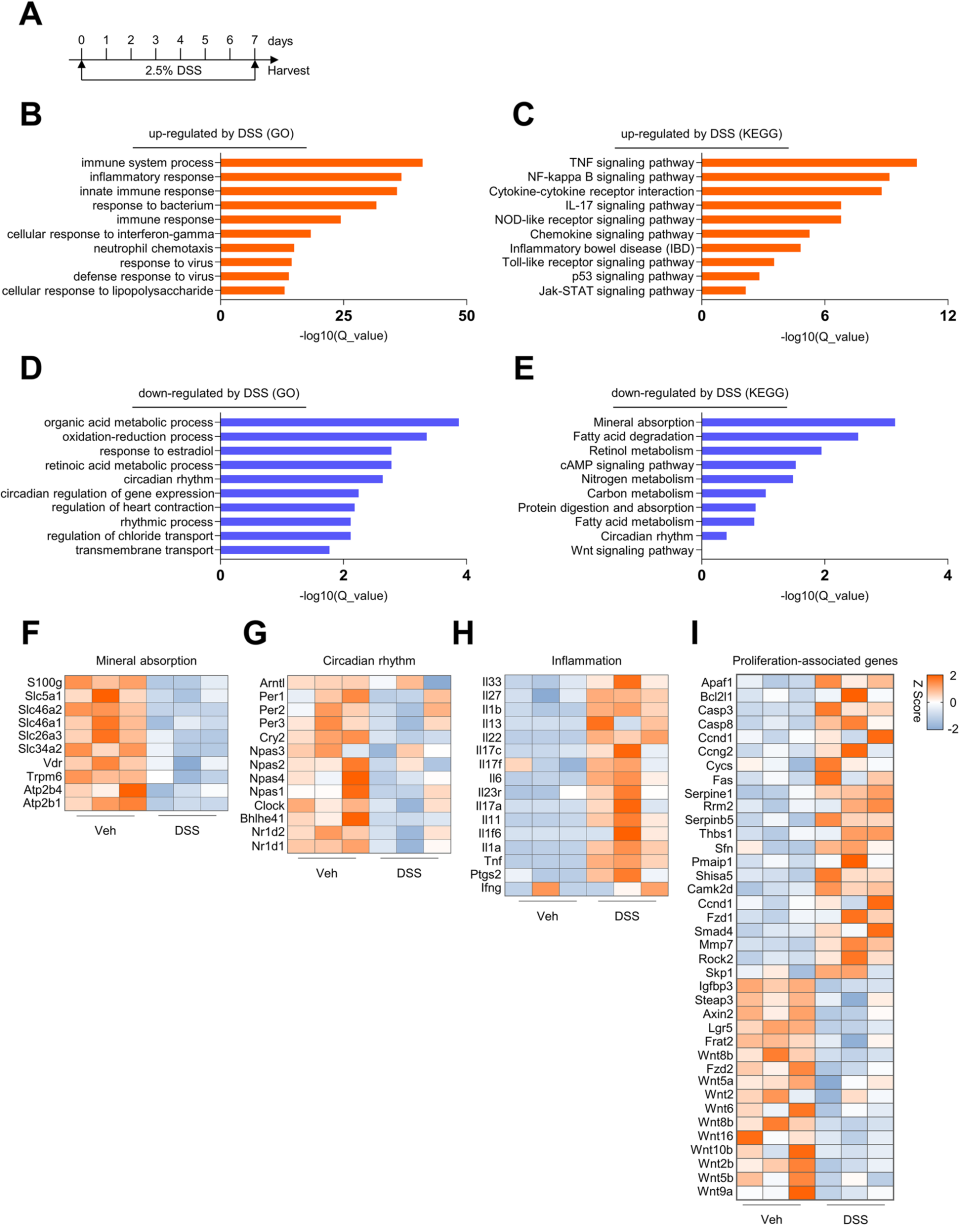
Gene expression profiling of rectal samples of colitis mice against normal mice. **A** Diagram showing the times for DSS treatment and sample collection. **B**, **C** GO and KEGG pathway analyses showing up-regulated gene clusters. **D**, **E** GO and KEGG pathway analyses showing down-regulated gene clusters. **F**–**I** Heatmaps of mineral absorption, circadian rhythm, inflammation, and proliferation-related genes, *n*=3 per group

Both BBR and SASP have been shown to inhibit NF-κB to suppress inflammation [38, 57]. The above findings suggest that BBR may have additional targets. To test this, we analyzed the gene expression profiles of rectal samples of normal mice, colitis mice, normal mice receiving BBR, and colitis mice receiving BBR. The RNA was isolated from the rectal samples and used for bulk RNA-seq. We first compared the colitis samples to normal samples (Fig. 2A). GO analysis revealed an increase in the expression of genes in immune response, inflammatory response, innate immunity, and immune response to bacteria or virus in the samples of colitis mice (Fig. 2B), and KEGG analysis revealed enrichment in TNFα, NF-κB, cytokine-receptor interaction, IL17, NOD, IBD, TLR, p53, and the Jak-Stat pathway genes (Fig. 2C). These results validated the inflammation feature of the colitis model. On the other hand, GO analysis revealed down-regulated expression of genes in transmembrane and chloride transport, organic acid and retinoic acid metabolism, circadian rhythms, and circadian regulation of gene expression (Fig. 2D), and KEGG analysis uncovered down-regulated expression of mineral absorption, protein, carbon, and fatty acid degradation and absorption, and circadian rhythm genes (Fig. 2E). These results suggest that colitis is associated with suppression of metabolism, mineral absorption, and circadian gene expression in addition to sustained inflammation.

Analysis of the genes in mineral absorption, circadian rhythms, and inflammation confirmed the altered expression of many genes in these pathways or modules in colitis samples (Fig. 2F–H). Intriguingly, we found a decrease in the expression of Wnt-β-Catenin pathway genes and Wnt molecules, and an increase in pro-proliferation genes such as cyclin D and G and anti-proliferation genes such as Caspase 3 and 8 and Fas (Fig. 2I). These results suggest that colitis has complex effects on proliferation of the cells in the colorectal tract.

### Gene expression profiling revealed pleiotropic effects of BBR on colitis

**Fig. 3:**
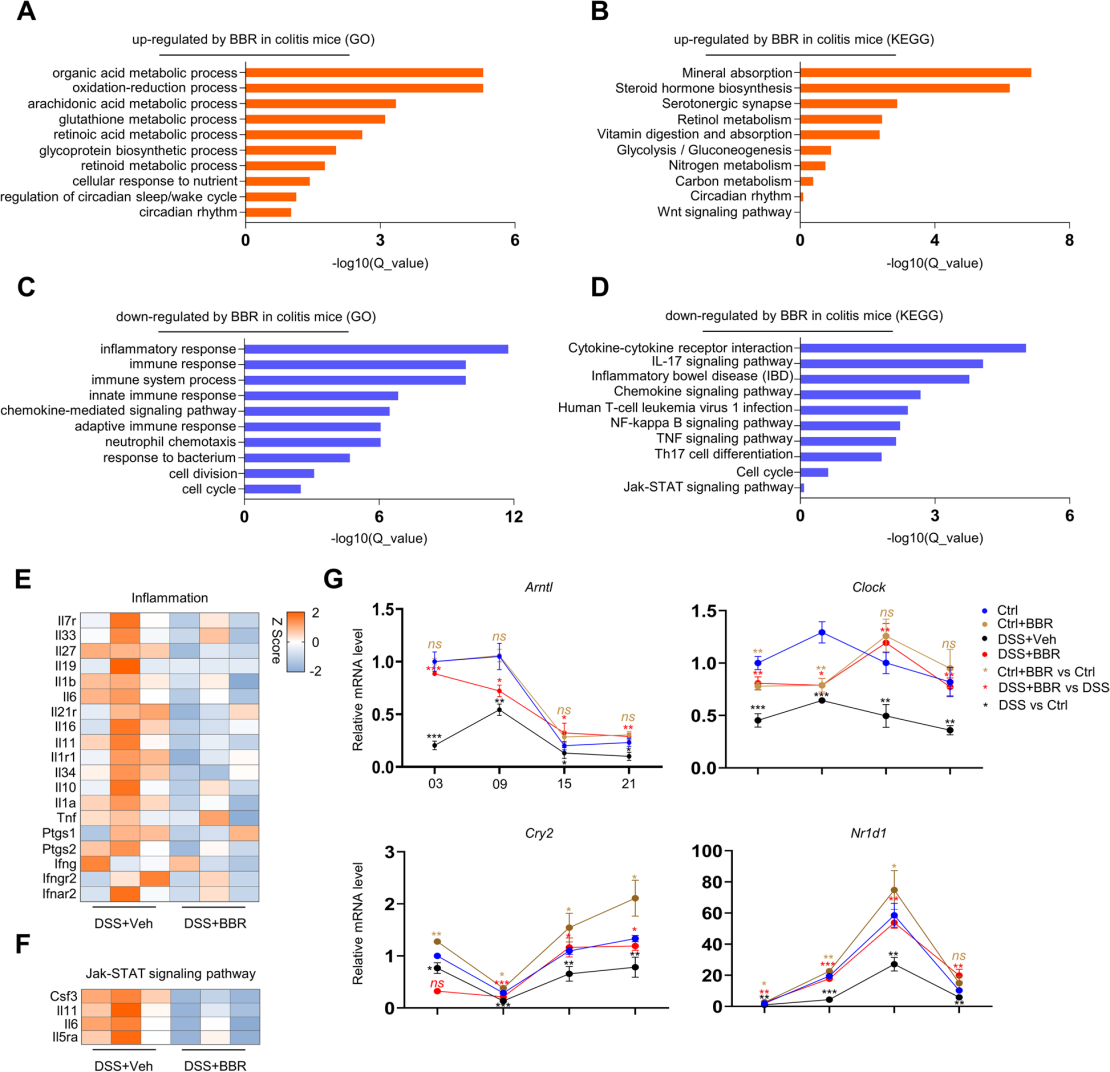
Gene expression profiling of rectal samples of BBR-treated colitis mice. **A**, **B** GO and KEGG pathway analyses showing up-regulated gene clusters by BBR in colitis mice. **C**, **D** GO and KEGG pathway analyses showing down-regulated gene clusters by BBR in colitis mice. **E**, **F** Heatmaps of inflammation-related and Jak-Stat signaling pathway genes, *n*=3 per group. **G** qPCR analysis of circadian rhythm genes in rectal samples of normal mice, colitis mice, normal mice receiving BBR, and colitis mice receiving BBR, which were collected at different time points of the day, *n*=3 per group. Data are presented as means ± SEM in **G**. Two-way ANOVA with Fisher’s LSD post hoc analysis (*α* = 0.05) was applied in **G**. * *p*<0.05, ** *p*<0.01, *** *p*<0.001. *p*<0.05 was considered as statistically significant

We then compared the gene expression profiles of rectal samples of colitis mice and colitis mice treated with BBR (Fig. 1A). GO analysis revealed that BBR increased the expression of genes in organic acid transport, oxidation-reduction, cell response to nutrient, and circadian genes in colitis mice (Fig. 3A), and KEGG analysis revealed that BBR promoted the expression of genes in mineral absorption, steroid hormone and retinol metabolism, and carbon metabolism, as well as circadian rhythm genes to a lesser extent (Fig. 3B). On the other hand, GO analysis showed that BBR suppressed the expression of genes in innate and adaptive immune response, inflammation, chemokine signaling pathway, chemotaxis, and cell response to bacteria (Fig. 3C), and KEGG analysis revealed that BBR suppressed the expression of genes in the pathways of cytokine-receptor, IL17, NF-κB, TNFα, and chemokine signaling (Fig. 3D). Further analysis confirmed that BBR suppressed the expression of many genes in inflammation and the Jak-Stat pathway (Fig. 3E, F). These results indicate that BBR has profound anti-inflammation effects and moreover, it rescues the expression of genes in circadian rhythm and mineral absorption.

We also compared the RNA-seq data of rectal samples of normal mice and normal mice treated with BBR (Fig. S3). GO analysis revealed that BBR up-regulated the expression of genes in a negative regulator of growth, protein folding, circadian genes, and retinol metabolism, and KEGG analysis showed that BBR up-regulated the expression of genes in mineral absorption, protein folding, amino acid metabolism, and circadian rhythms (Fig. S3A–E). On the other hand, GO analysis showed that BBR inhibited the expression of the cell cycle, cell division, and DNA repair genes, and KEGG analysis revealed that BBR suppressed cell cycle, cancer, and p53 signaling pathway genes (Fig. S3A–E). Further analysis confirmed that BBR increases the expression of several mineral absorption and circadian rhythm genes (Fig. S3E, G). The fact that BBR showed similar effects on gene expression profiles in normal mice and colitis mice suggests that many of the effects of BBR are not secondary to inflammation suppression.

The above studies indicate that colitis and BBR profoundly affect the expression of mineral absorption-related genes. qPCR analysis confirmed the reduction in the expression of several mineral absorption-related genes including *Atp2b1* and *Atp2b4*, which were rescued by BBR (Fig. S4A). Western blot analysis of the colon samples confirmed a reduction in plasm membrane Ca^2+^ pump proteins (PMCA), encoded by *Atp2b1*, *Atp2b2*, *Atp2b3*, and *Atp2b4* and recognized by the same antibody, in colitis mice, which was rescued by BBR (Fig. S4B). These results suggest that BBR may help mineral absorption in colitis mice.

Since disruption of circadian rhythms increases IBD risks in human and mice [17], we compared the colon samples of colitis mice and colitis mice treated with BBR at different time points of the day: 03 (3am), 09 (9am), 15 (3pm), 21 (9pm), and found no significant difference in colon length or histology scores among the four groups (Fig. S5A–C), suggesting that the colitis pathology itself does not show day-night rhythm. We also compared the expression of several key circadian genes including *Arntl*, *Clock*, *Cry1*, *Cry2*, *Per1*, *Per2*, *Per3*, *Nr1d1*, and *Npas2* in colon samples collected at 03, 09, 15, and 21 of normal mice, colitis mice, normal mice receiving BBR, and colitis mice receiving BBR. We found that expression of many circadian genes showed day-night rhythms (Fig. 3G and Fig. S5D). In general, DSS greatly suppressed the expression of these circadian genes, which were largely rescued by BBR (Fig. 3G and Fig. S5D). On the other hand, DSS and BBR only slightly affected the day-night rhythms of circadian gene expression (Fig. 3G and Fig. S5D). These results suggest that circadian genes are targeted by BBR in colitis mice.

### Transcriptome analysis confirmed that BBR activated Wnt and circadian gene expression compared to SASP

We also conducted RNA-seq on colon samples of normal mice, colitis mice, normal mice receiving SASP, and colitis mice receiving SASP. We found that compared to colitis samples, SASP suppressed the expression of genes regulating inflammation and chemotaxis (*Ptgs2*, *IL1b*, *IL6*, *Ccl2*, *Ccl3*, *Cxcl1*, *Cxcl2*, *Cxcl3*, *Cxcl5*, and *Cxcl12*), oxidative phosphorylation, IL17 signaling, and NF-kB signaling; and up-regulated genes regulating protein folding, RNA splicing, DNA replication and repair, amino acid metabolism, and some immune response genes (*Irf1*, *H2-Q4*, *H2-T2*3, and *Pamb8*) (Fig. S6A–D). Overall, the findings support the anti-inflammation activity of SASP. Moreover, we compared the transcriptomes of BBR-treated colitis samples and SASP-treated colitis samples and found that BBR up-regulated the expression of genes involved in ECM, Calcium ion transport, wound healing, stromal cells, Wnt, Akt, MAPK, and circadian genes and suppressed the expression of genes involved in cell cycle, DNA repair, IBD, immune response (Fig. S6E–J). These results further support that BBR and SASP have different actions in colitis treatment and that BBR increases the expression of circadian genes and Wnt molecules.

### BBR suppressed macrophages and granulocytes but not T cells in colitis models

**Fig. 4:**
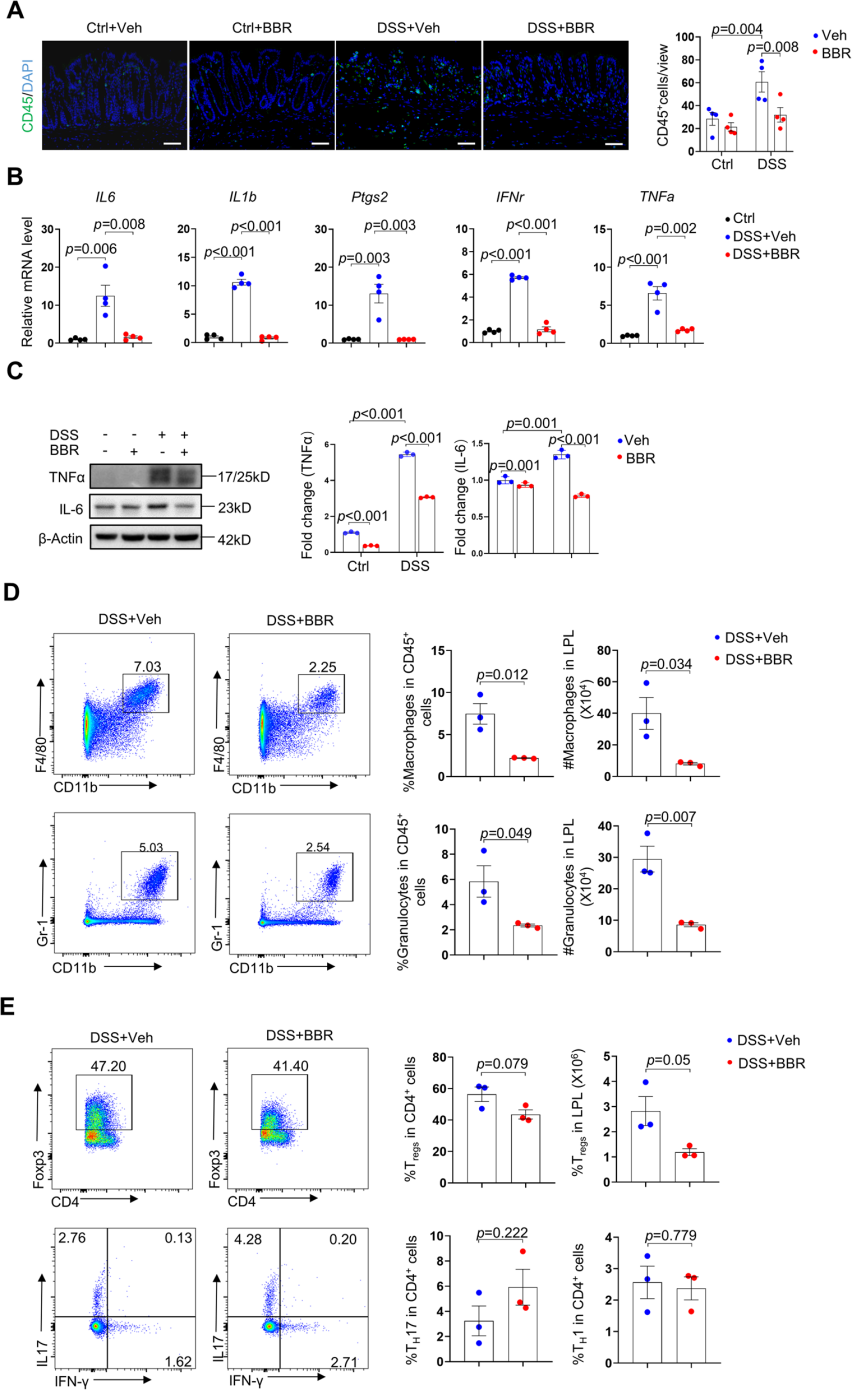
The effects of BBR on immune responses in colitis mice. **A** Immunostaining showed CD45^+^ cells in colon samples of normal mice, colitis mice, normal mice receiving BBR, and colitis mice receiving BBR, *n*=4 per group. Scale bars, 50 μm. Each dot represented the average of at least five views per sample. Right panel: Quantitation results. **B** Expression of *Il6*, *Il1b*, *Ptgs2*, *IFNγ*, and *TNFα* in rectal samples of the four mouse groups, *n*=4 per group. **C** Western blot analysis revealed that the colon samples of colitis mice showed an increase in TNFα and IL6, which was suppressed by BBR. Right panels: quantitation data. *n*=3. **D** Flow cytometric results showed the percentages and numbers of macrophages and granulocytes in colorectal samples of the four mouse groups. Right panels: Quantitation results. *n*=3 per group. **E** Flow cytometric analysis of T_H_1, T_H_17, and T_reg_ cells in colorectal samples of the four mouse groups. Right panels: Quantitation results. *n*=3 per group. Data are presented as means±SEM in **A**–**E**. Unpaired two-tailed Student’s *t* test was applied in **B**–**E**, two-way ANOVA with Fisher’s LSD post hoc analysis (*α* = 0.05) was applied in **A**. *p*<0.05 was considered as statistically significant. Abbreviations: DAPI, 4′,6-diamidino-2-phenylindole; LPL, lamina propria lymphocytes

Our RNA-seq data indicate that BBR suppressed inflammation. To verify this, we immunostained rectal sections for immune cell marker CD45 and found the signals were increased in DSS-treated samples compared to control samples, which were suppressed by BBR (Fig. 4A). qPCR analysis revealed that BBR suppressed the expression of *IL6*, *IL1β*, *Ptgs2*, *IFNγ*, and *TNFα* in rectal samples (Fig. 4B). Western blot analysis of TNFα and IL6 in colon samples confirmed an increase in these two proteins in colitis mice, which was suppressed by BBR (Fig. 4C). We then designed a panel to identify and quantify all leukocyte populations in the colorectal tract by flow cytometry and found that BBR inhibited the activation/infiltration of macrophages and granulocytes but not T cells (Fig. 4D, E and Fig. S7A, B), consistent with previous studies reporting suppression of macrophages but inconsistent with previous studies reporting suppression of T cells by BBR [42, 45, 46]. Moreover, it has been shown that BBR may inhibit inflammation via NF-κB and AMPKs [39]. Our RNA-seq data show that colitis activated whereas BBR suppressed Jak-Stat3 signaling (Fig. 2C, Fig. 3D, and F), a critical regulator of cytokine expression and inflammation [58], consistent with previous studies showing that Stat3 activation might be affected by BBR [31, 59].

### BBR promoted ISC proliferation and epithelial repair in colitis models

**Fig. 5:**
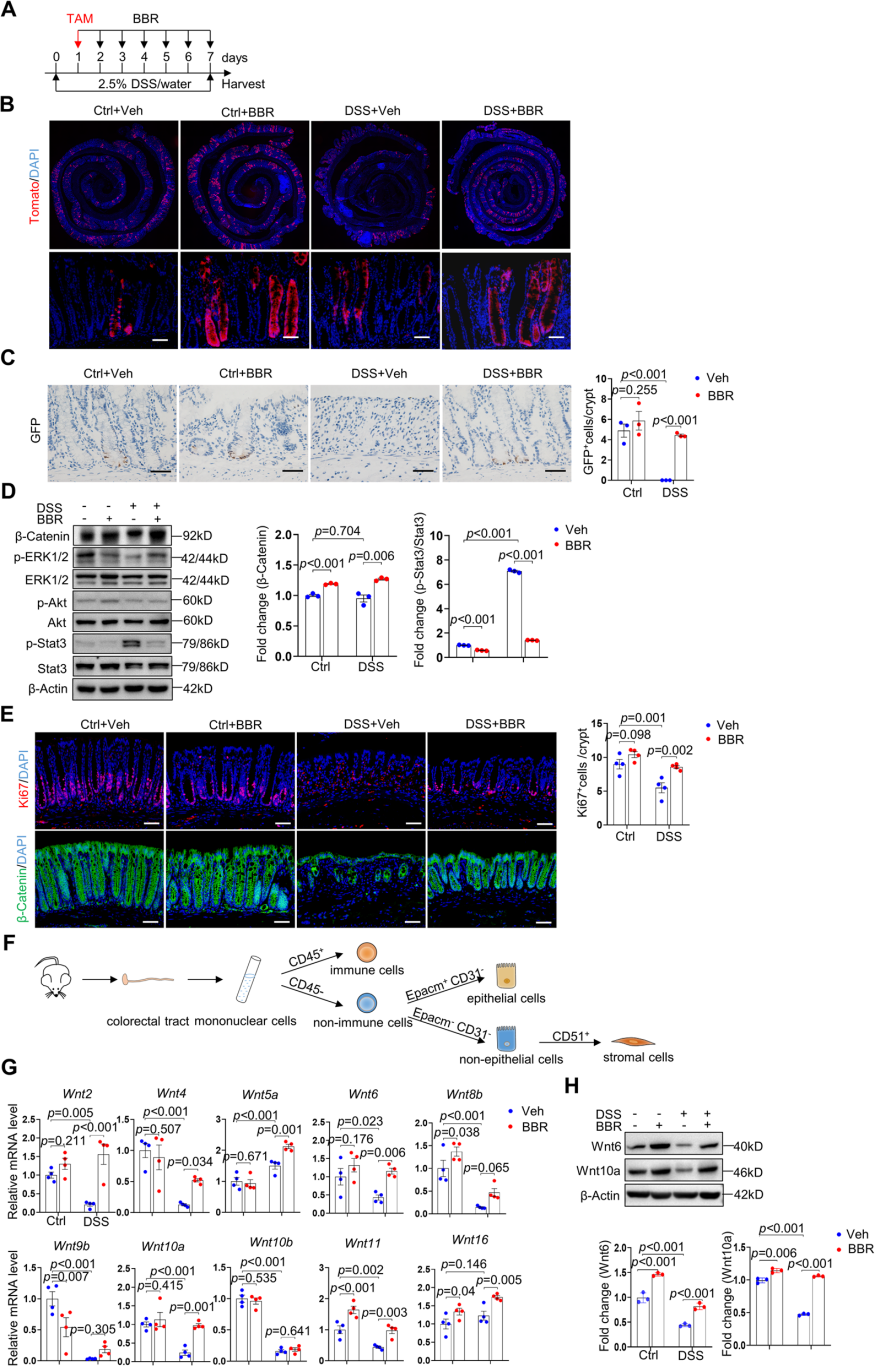
BBR promoted stromal expression of Wnts and ISC-mediated epithelial repair. **A** Diagram showing the times of DSS, BBR, and TAM treatment and colorectal sample collection. **B** Tracing of *Lgr5*^+^ cells in mouse colorectum. The samples of *Lgr5-CreERT; tdTomato* adult mice were sectioned and stained with DAPI. Scale bars, 50 μm. **C** DAB staining showed the number of Lgr5^+^ cells stained with GFP in the four mouse groups, *n*=3 per group. Scale bars, 50 μm. Each dot represented the average of at least five views per sample. Right panel: Quantitation results. **D** WB analysis of activation of β-Catenin and other signaling molecules in the colon samples of the four mouse groups. *n*=3 per group. **E** Immunostaining showed *Ki67*^+^ cells and activation of β-Catenin on rectal samples of normal mice, colitis mice, normal mice receiving BBR, and colitis mice receiving BBR. Scale bars, 50 μm. Each dot represented the average of at least five views per sample. *n*=4 per group. Right panels: Quantitation results. **F** Schematic diagram showing the sorting strategy of colorectal immune, epithelial, and stromal cells. **G** qPCR analysis of various *Wnt* molecules in stromal cells of the four mouse groups, *n*=3 per group. H Western blot analysis revealed that the colon samples of colitis mice showed a decrease in Wnt6 and Wnt10a, which was rescued by BBR. Right panels: quantitation data. *n*=3. Data are presented as means±SEM in (**C**-**H**). Two-way ANOVA with Fisher’s LSD post hoc analysis (*α* = 0.05) was applied in (**C**-**H**). *p*<0.05 was considered as statistically significant. Abbreviation: TAM, tamoxifen

Our RNA-seq data indicate that BBR affects not only cell division and cell cycle genes but also anti-proliferation genes (Fig. 2 and Fig. S3). Colorectal epithelia undergo rapid turnover, driven by Lgr5^+^ ISCs. To test the effects of BBR on ISCs, we induced colitis in *Lgr5-CreERT-GFP; Rosa-tdTomato* mice [5], which were treated with BBR. The genotypes of the mice were verified with PCR analysis of the genomic DNA (Fig. S8). While tamoxifen (TAM) administration led to Tomato labeling of the descendent cells of Lgr5^+^ ISCs, GFP staining detected ISCs (Fig. 5A). We found that BBR significantly increased the number of Lgr5^+^ ISCs and promoted the renewal activity of Lgr5^+^ ISCs, manifested by an increase in the number of GFP^+^ ISCs and Tomato^+^ epithelial cells, respectively (Fig. 5B, C). These results indicate that BBR promotes colorectal epithelial repair in experimental colitis models. Moreover, we found that in non-colitis mice, BBR modestly increased the colorectal epithelial turnover but not the number of GFP^+^ ISCs (Fig. 5B, C), suggesting that the epithelial phenotype is not secondary to inflammation suppression by BBR.

### BBR promoted the expression of Wnt genes in colorectal stromal cells

The activity of ISCs is controlled by their niche, especially the resident stromal cells [6], which secrete Wnts, BMPs, Notch, and other signaling molecules to regulate ISC proliferation and differentiation. In particular, Wnts are critical for ISC proliferation [60]. Western blot analysis of major mitogenic pathways in rectal homogenates revealed that β-Catenin, a downstream transcription factor stabilized by Wnts, was greatly elevated by BBR treatment in colitis samples (Fig. 5D). On the other hand, BBR treatment did not affect ERK and Akt activation (Fig. 5D). We immunostained the rectal sections for β-Catenin and found that BBR increased β-Catenin signals in epithelial cells, associated with an increase in Ki67^+^ proliferating cells (Fig. 5E). These results suggest that Wnt-β-Catenin may play an important role in restitution of colorectal epithelia. We also detected increased p-Stat3 levels in the samples of colitis mice, which was suppressed by BBR (Fig. 5D). These results, together with our RNA-seq results (Fig. 2C, Fig. 3D, and F), suggest that Stat3 pathway plays a role in BBR-induced suppression of inflammation.

A recent study reported that BBR activated Wnt-β-Catenin signaling in colonic epithelial cells to protect the mucosal barrier and blocking the activation of this pathway with FH535, a β-Catenin/Tcf inhibitor, diminished the barrier-protection and immune-suppression activities of BBR [41]. We tested whether BBR directly acted on epithelial cells and activated the β-Catenin pathway using colonic epithelial cell line HCT116. We treated the cells with different doses of BBR and found that BBR activated ERK and Akt to minimal extents; however, it decreased the protein levels of β-Catenin (Fig. S9). These results suggest that increased activation of β-Catenin in colorectal epithelial cells of BBR-treated colorectal samples is unlikely to be cell-autonomous. Instead, BBR may act on other cells to stimulate the synthesis and secretion of Wnt molecules, which then activate β-Catenin in epithelial cells to promote the expression of tight junction proteins. Certainly, this warrants further investigation.

The colonic tissues contain immune cells (CD45^+^), endothelial cells (CD31^+^), and stromal cells besides epithelial cells (EpCAM^+^). Stromal cells do not express CD45, CD31, or EpCAM. Recent studies have shown that stromal cell express CD51 [61]. We isolated stromal cells (CD45^-^CD31^-^EpCAM^-^CD51^+^) from colorectal mononuclear cells by excluding immune cells, endothelial cells, and epithelial cells (Fig. 5F and Fig. S10A). We also analyzed the expression of stromal cell markers Vimentin and CD29 [62, 63] on sorted stromal cells by flow cytometry and found that more than 96.6% of cells were positive for the two markers (Fig. S10A). We then immunostained the sorted cells for Vimentin, EpCAM, CD31, and CD45 and found that all the sorted cells were positive for Vimentin but negative for EpCAM, CD31, and CD45 (Fig. S10B). These results verified the identity of the stromal cells.

qPCR analysis revealed that the stromal cells expressed many Wnt molecules (Fig. 5G). In DSS-induced colitis models, the expression of Wnt molecules including *Wnt2*, *4*, *6*, *8b*, *9b*, *10a*, *10b*, and *11* were suppressed in stromal cells, which was restored by BBR treatment (Fig. 5G), while expression of *Wnt5a* and *Wnt16* was not significantly affected by DSS or BBR and other Wnt molecules were not detected (Fig. 5G). Western blot analysis of the colon samples for Wnt6 and Wnt10a expression confirmed a reduction in these two proteins in colitis mice, which was rescued by BBR (Fig. 5H). Overall, these results suggest that BBR might induce ISC expansion via stromal cell-produced Wnt molecules, consistent with the fact that Lgr5 is a target of Wnt signaling and that Lgr5 activation drives ISC proliferation [60].

### Inhibiting Wnt secretion diminished the therapeutic effect of BBR

**Fig. 6:**
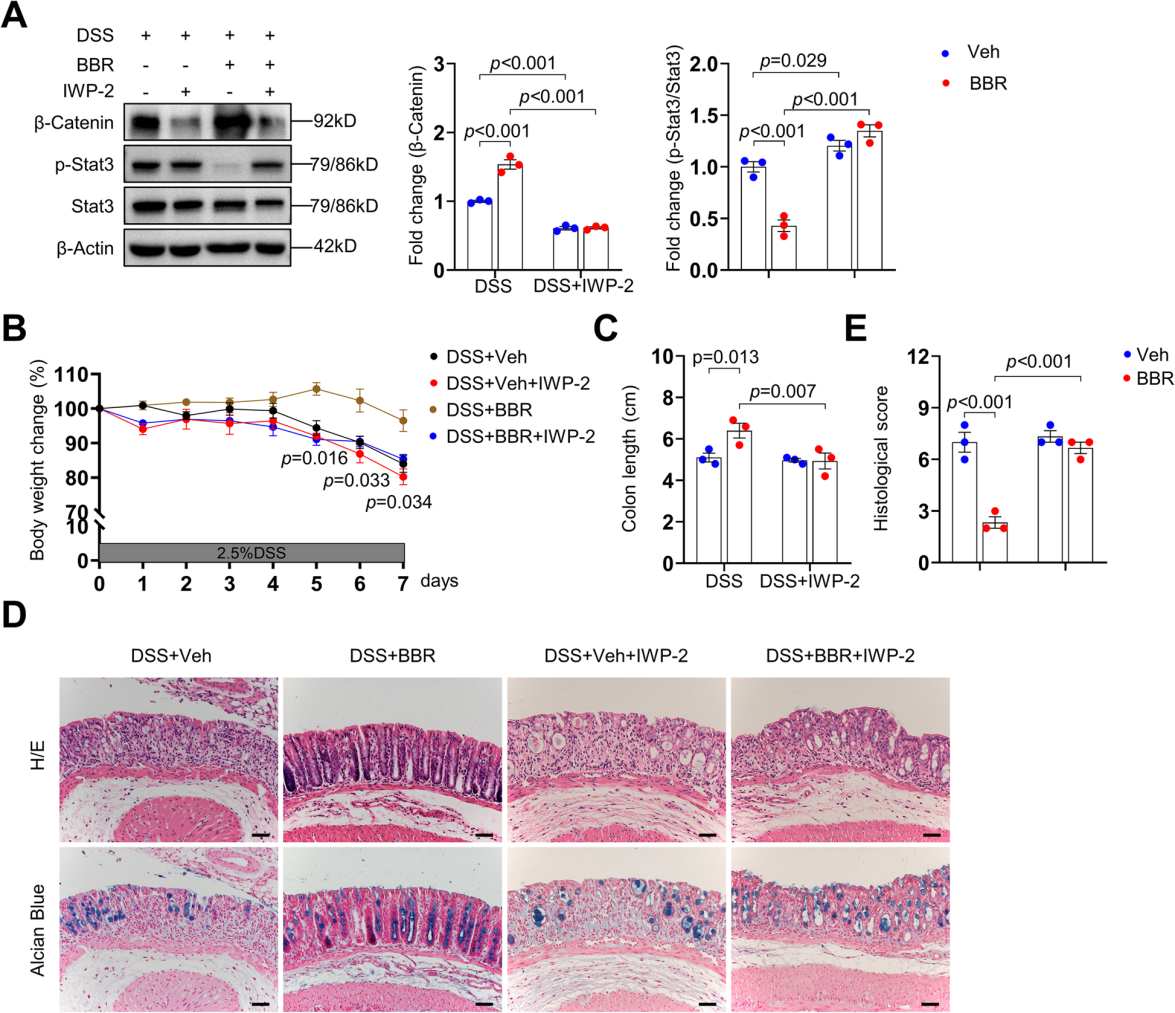
Inhibiting Wnt secretion dampened the therapeutic effects of BBR on colitis. **A** WB results showed activation of β-Catenin in rectal samples of colitis mice, colitis mice receiving IWP-2, colitis mice receiving BBR, and colitis mice receiving BBR and IWP-2, *n*=3 per group. **B–E** The body weights (**B**), colon length (**C**), and histological scores (**E**) were determined and H/E and Alcian Blue staining of rectal sections was performed (**D**). *n*=3 per group. Scale bars, 50 μm. Data are presented as means±SEM in **A**–**C** and **E**. Two-way ANOVA with Fisher’s LSD post hoc analysis (*α* = 0.05) was applied in **A**–**C** and **E**. *p*<0.05 was considered as statistically significant.

The above studies showed that Wnt molecules were down-regulated in DSS-induced colitis samples whereas BBR could restore their expression in resident stromal cells. To test the in vivo roles of Wnt-β-Catenin activation, we administrated IWP-2, an inhibitor of PORCN (Porcupine O-Acyltransferase) required for Wnt modification and secretion, to the mice via peritoneal injection at a dose that has been previously used [64]. We found that β-Catenin signals were significantly suppressed (Fig. 6A). Moreover, in the presence of IWP-2, the therapeutic effects of BBR on colitis mice including the body weight, the colon length, histological changes, the number of goblet cells, and the colitis score were largely diminished (Fig. 6B–E). The remaining effect is likely due to the immune suppression activity of BBR, as we found that IWP-2 did not affect Stat3 activation (Fig. 6A). Overall, these results suggest that Wnt secretion, likely by stromal cells, is required for BBR to execute its therapeutic effect on experimental colitis.

### BBR restored circadian gene expression in stromal cells

Our RNA-seq data indicate that colitis was associated with a decrease in the expression of various circadian rhythm genes, which was restored by BBR (Figs. 2 and 3). Previous studies have implicated circadian clock in bone marrow/peripheral macrophages as a regulator of IBD pathogenesis [8]. To determine the contribution of various colorectal cell types to the change in circadian gene expression, we isolated CD45^+^ immune cells, EpCAM^+^ epithelial cells, and CD51^+^ stromal cells from the colon of normal mice, colitis mice, normal mice receiving BBR, and colitis mice receiving BBR, via FACS sorting (Fig. 5F). We isolated RNA from these cells and performed qPCR. We found that expression of major circadian genes was not altered by colitis or by BBR treatment in epithelial cells (Fig. S11A). In immune cells, colitis was associated with a decrease in expression of *Per2* while the expression of other key circadian genes was not affected (Fig. S11B). However, BBR failed to rescue the expression of *Per2* in colitis mice (Fig. S11B). These results suggest that colitis and BBR only have a minor effect on the expression of circadian genes in epithelial cells and immune cells of the colorectal tract.

**Fig. 7:**
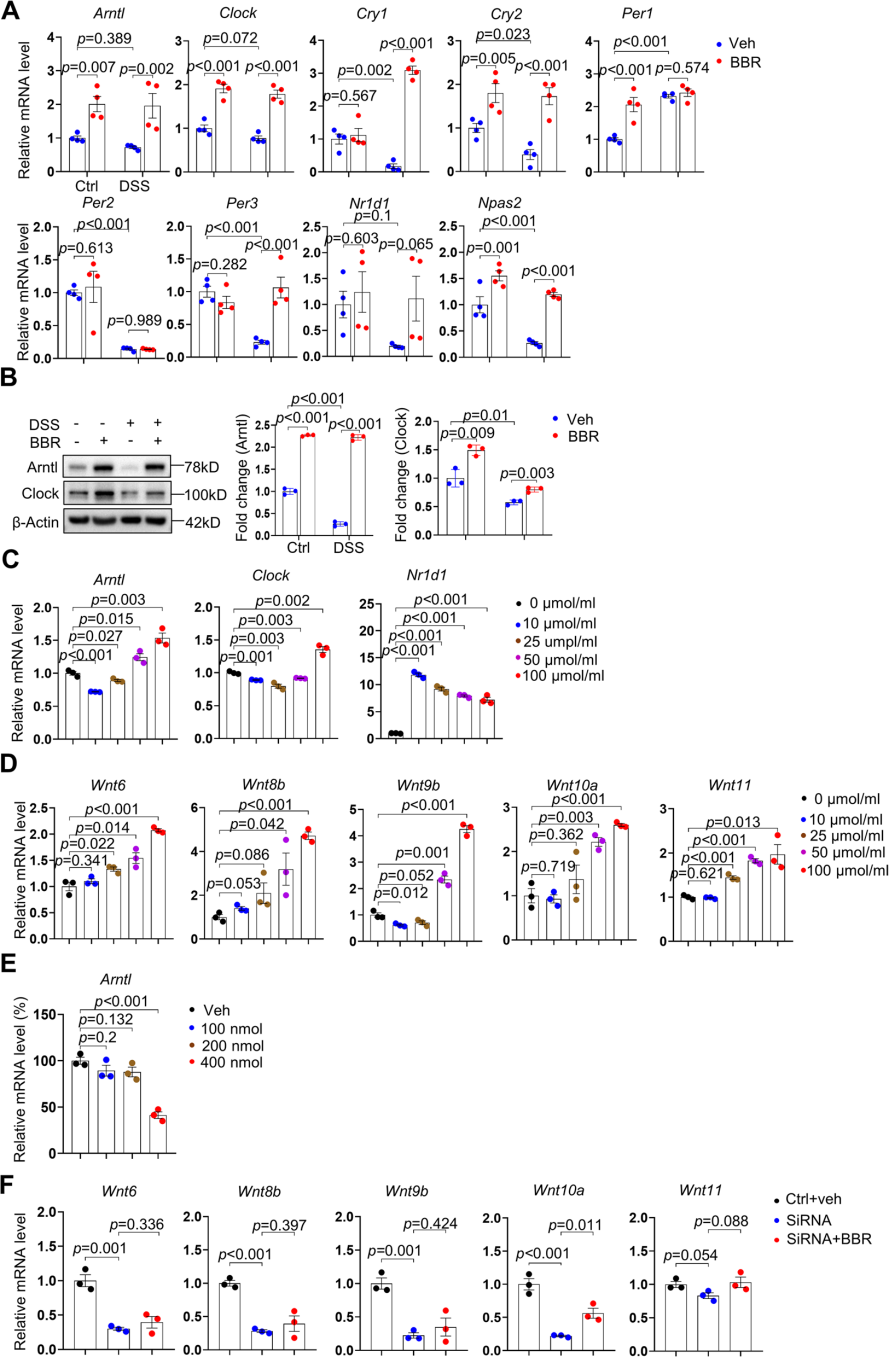
BBR promotes expression of Wnt genes via circadian rhythm. **A** qPCR analysis of various circadian genes in stromal cells from normal mice, colitis mice, normal mice receiving BBR, and colitis mice receiving BBR, *n*=4 per group. **B** Western blot analysis revealed that the colon stromal cells of the colitis mice showed a decrease in Arntl and Clock, which was rescued by BBR. Right panels: quantitation data. *n*=3. **C** qPCR analysis of *Arntl*, *Clock*, *Nr1d1* in cultured stromal cells treated by different dose of BBR, *n*=3 per group. **D** qPCR analysis of *Wnt6*, *Wnt8b*, *Wnt9b,Wnt10b*, and *Wnt11* in cultured stromal cells treated with different dose of BBR, *n*=3 per group. **E** Expression of *Arntl* in cultured stromal cells treated by different concentrations of *Arntl siRNA*, *n*=3 per group. **F** qPCR analysis of *Wnt6*, *Wnt8b*, *Wnt9b*, and *Wnt10b* but not *Wnt11* in *Arntl siRNA* knock-down cells (400 nmol) compared to control. *n*=3 per group. Data are presented as means±SEM in **A**–**F**. Unpaired two-tailed Student’s *t* test was applied in **C**–**F**, two-way ANOVA with Fisher’s LSD post hoc analysis (*α* = 0.05) was applied in **A** and **B**. *p*<0.05 was considered as statistically significant

On the contrary, we found that in colorectal stromal cells, colitis was associated with decreases in the mRNA levels of *Arntl*, *Clock*, *Cry1*, *Cry2*, *Per2*, *Per3*, *Nr1d1*, *Npas2*, and BBR restored the expression of these genes except *Per2* (Fig. 7A). Furthermore, we found that BBR increased the expression of circadian genes including *Arntl*, *Clock*, *Cry2*, *Per1*, and *Npas2* in stromal cells of normal mice (Fig. 7A). Western blot analysis confirmed a reduction in *Arntl* and *Clock* protein levels in the stromal cells of the colitis mice, which was rescued by BBR (Fig. 7B). These results suggest that colitis and BBR mainly affect the circadian rhythm in resident stromal cells rather than epithelial and immune cells.

### Circadian rhythms play a role in BBR-induced Wnt gene expression

Recent studies have shown that circadian rhythms in ISCs are controlled by niche cells during regeneration [13, 65]. To determine whether the circadian rhythm is involved in BBR-induced Wnt expression in stromal cells, we used the primary colorectal stromal cells as a model. In cultured cells, we found that BBR promoted the expression of circadian rhythm genes including *Arntl*, *Clock*, and *Nr1d1* but no other genes (Fig. 7C). Moreover, BBR promoted the expression of various *Wnt* genes including *Wnt6*, *8b*, *9b*, *10a*, and *11* (Fig. 7D). These results suggest that BBR directly acts on stromal cells. Since not all circadian genes or Wnt genes are up-regulated by BBR in cultured stromal cells, we speculate that BBR also regulates the expression of circadian genes and *Wnt* genes in non-cell-autonomous manners.

We then knocked down the main circadian gene *Arntl* in cultured stromal cells with siRNA (Fig. 7E). We found that knock-down of *Arntl* inhibited the expression of *Wnt6*, *8b*, *9b*, and *10a* but not *Wnt11* in cultured stromal cells (Fig. 7F). Moreover, in *Arntl* knockdown cells, BBR failed to increase the expression of *Wnt6*, *8b*, *9b*, and *10a* but not *Wnt11* (Fig. 7F). Taken together, these results suggest that BBR promotes the expression of a number of *Wnt* genes in a circadian-dependent manner in stromal cells.

## Discussion

IBDs are common diseases and the current treatments mainly target immune cells or inflammatory cytokines, with limited success. Early studies have tested targeting epithelial cells, e.g., by activating EGF or TGFβ signaling, to treat colitis, yet, those studies failed to meet the expectation due to the mild efficacy and side effects of the treatments. Here, we show that BBR, a natural product, executes its therapeutic effects on colitis by suppression of inflammation and promoting epithelial repair via activating ISCs. These findings support BBR as a drug candidate to treat patients with colitis.

We show that BBR suppresses the activation of macrophages and granulocytes in colitis mouse models, accompanied by decreases in the production of inflammatory cytokines including IL1β, TNFα, and IL6, yet, T cells are not significantly affected. These findings are consistent with the reported effect of BBR on macrophages but not consistent with the reported effects on T cells [42, 45, 46]. Although BBR has been shown to inhibit NF-κB and AMPK in immune cells [66], we find that BBR also inhibits the activation of Jak-Stat3 signaling, a well-established regulator of cytokine production and inflammation [58]. Overall, our studies support the anti-innate immunity effects of BBR and implicate Stat3 as a mediator in its action.

IBD patients often show disrupted crypt structure in the colorectal tract, especially ulcerative colitis patients. Importantly, we show that BBR promotes ISC activation and the repair of the colorectal epithelia. We find that resident stromal cells expressed several Wnt molecules, and moreover, BBR promotes the expression of the Wnt molecules in these cells. Accordingly, we observe an increase in β-Catenin activation in BBR-treated epithelial cells, accompanied by increased ISC activation. Furthermore, we find that inhibition of Wnt secretion dampens the therapeutic effect of BBR. These results suggest that ISC-mediated epithelial repair is a key factor in colitis treatment.

In this study, our gene expression profiling and quantitative PCR assays reveal that the expression of circadian clock genes is suppressed in experimental colitis, mainly in resident stromal cells. Recent studies suggest that macrophages show circadian rhythms in colitis mouse models [24, 67]. BBR might act on Rev-ERB and mice with Rev-ERbα ablation show more severe colitis, likely via enhanced NF-κB activation in macrophages [23, 67]. Intriguingly, we show that only the expression of *Per2* is suppressed in the colorectal immune cells in colitis mice, which was not affected by BBR, suggesting that circadian rhythms in colonic immune cells are not a major target of BBR. This discrepancy may be caused by analyzing colorectal immune cells in our study and analyzing circulating immune cells in the other study [67]. In contrast to epithelial and immune cells, we uncover decreased expression of a wide-spectrum of circadian genes in colonic stromal cells of colitis mice. The expression of circadian genes shows a day-night rhythm in normal colon samples, which was suppressed in colitis mice. BBR rescues circadian gene expression, although the day-night rhythm is only slightly affected. Overall, these results suggest that BBR may execute its therapeutic effects via affecting circadian gene expression in resident stromal cells rather than immune cells or epithelial cells.

**Fig. 8:**
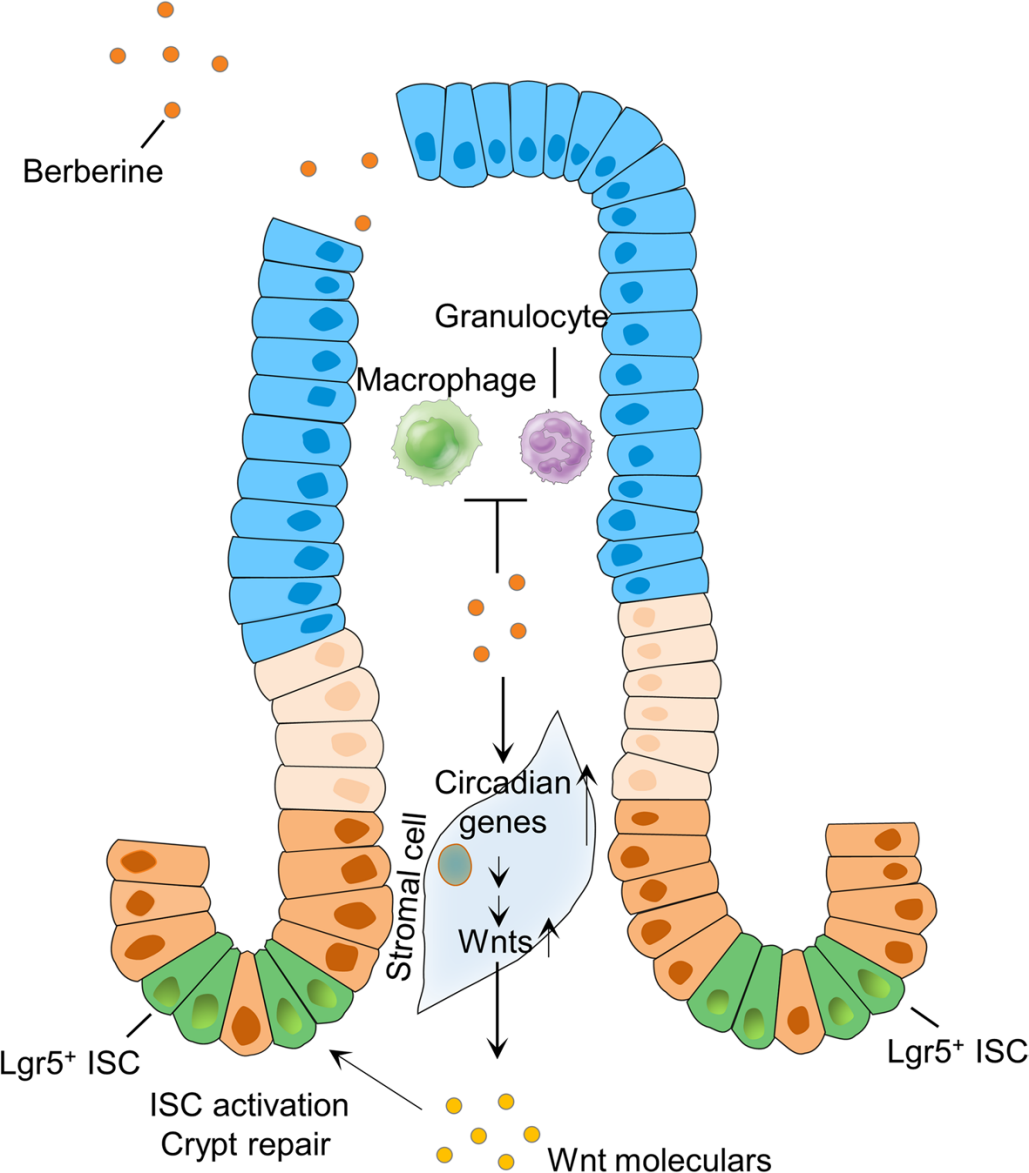
Schematic depiction of the molecular mechanism by which BBR promotes colonic epithelial repair via resident stromal cells and ISCs in colitis treatment

We further show that the circadian clock regulates BBR-induced Wnt production in stromal cells. Previous studies have reported that ISCs show circadian rhythms during regeneration but not under physiological conditions [68, 69]. Here, we show that colitis is associated with decreased expression of circadian rhythm genes in resident stromal cells, which leads to reduced expression of Wnt molecules. These results suggest that the circadian clock in stromal cells affects ISC activities via controlling the synthesis of Wnt molecules (Fig. 8), thus revealing a novel mechanism by which the circadian clock contributes to colitis pathogenesis.

How does BBR affect the expression of clock genes in stromal cells? Since gut microbiota directly regulate circadian rhythms and BBR has been shown to alter the composition of intestinal microbiota [70], one possibility would be that BBR acts on microbes, which in turn regulate circadian gene expression in stromal cells. However, we find that BBR affects the expression of several key circadian genes in cultured stromal cells, suggesting that BBR may directly regulate circadian gene expression as well. In addition, previous studies have shown that BBR activates AMPK [71], which is an important regulator of circadian genes [72]. We speculate that BBR may also control circadian gene expression via AMPK, this certainly warrants further investigation.

While colitis pathogenesis studies have mainly focused on immune cells and microbiota, our recent studies have shown that stromal cell-generated PGE_2_ plays a critical role in suppressing colitis development and IBD patients show a reverse correlation between the levels of Cox2 (an enzyme to synthesize PGE_2_) expression in colorectal stromal cells and severity of the disease [73]. It is likely that loss of anti-inflammation activity in resident stromal cells contributes to the pathogenesis of IBDs. Recent single-cell profiling also reveals that stromal cells might even gain pro-inflammatory activities in IBD [62, 74]. Here, we show that BBR targets colon resident stromal cells to increase the expression of Wnt molecules, which in turn activate ISCs to promote epithelial regeneration. Overall, these results underscore the importance of resident stromal cells in the pathogenesis of colitis as well as the treatment of colitis.

## Conclusions

Our study shows that berberine is more effective in treating experimental colitis than sulfasalazine. BBR suppresses the activation of innate immune cells and promotes intestinal stem cell expansion and colonic epithelial repair in colitis models. Mechanistically, BBR up-regulates the expression of Wnt molecules in resident stromal cells, the ISC niche, via circadian rhythms, to activate ISCs (Fig. 8). These findings suggest that the stromal niche/ISC duo is a feasible target for IBD treatment and that the dual effects of BBR in suppressing inflammation and promoting epithelial repair make it a strong candidate to treat IBD patients.

## Methods

### Chemicals and reagents

Berberine chloride (purity ≥98%) was purchased from Shanghai Meryer Chemical Technology Co., Ltd (Shanghai China) and Sichuan Good Doctor Panxi Pharmaceutical Co., LTD. (Xichang China). Dextran sulfate sodium (DSS) was purchased from MP Biomedicals (MW; 36000-50000, MP Biomedicals, Solon, OH, USA); Sulfasalazine was from Shanghai Fuda Pharmaceutical Co. Ltd (Shanghai China). Tamoxifen was purchased from Sigma (USA). Lipofectamine^TM^2000 was purchased from Invitrogen (USA). Berberine, sulfasalazine, and DSS were dissolved in water.

### Mice

All mouse works followed the recommendations of the National Research Council Guide for the Care and Use of Laboratory Animals, with the protocols approved by the Institutional Animal Care and Use Committee of Shanghai, China [SYXK(SH)2011-0112]. Male C57BL/6 mice, age 8~10 weeks, weight 23~25g, were obtained from the Shanghai Model Organisms Center, Inc. *Lgr5-GFP-CreERT* mice were generated in Hans Clever’s lab and *Rosa-tdTomato* mice were purchased from The Jackson Laboratories. The mice were maintained in the SPF animal facility of Shanghai Jiao Tong University. The colon samples used in this study were collected starting from 9am unless designated otherwise.

#### Genotyping of Lgr5-GFP-CreERT; Rosa-tdTomato mice

The tail DNA was used for genotyping. Standard PCR reactions were conducted for genotyping. The following primers were used: Lgr5-CreERT 8060:5′-CTGCTCTCTGCTCCCAGTCT -3′, Lgr5-CreERT 8061:5′-ATACCCGGTGAACAGCTC -3′, Lgr5-CreERT 9402:5′-CACCCCGGTGAACAGCTC -3′. Rosa-tdTomato MR9020:5′-AAGGGAGCTGCAGTGGAGTA-3′, Rosa-tdTomato MR9021:5′-CCGAAAATCTGTGGGAAGTC-3′, Rosa-tdTomato MR9103:5′-GGCATTAAAGCAGCGTATCC-3′, Rosa-tdTomato MR9105:5′-CTGTTCCTGTACGGCATGG-3′. The amplified DNA was electrophoresed onto 3% agarose gel.

### Induction of colitis and treatment regimens

For induction of the colitis model, adult male mice were given 2.5% DSS in drinking water for 7 days. For treatment, the mice were divided into four groups: normal mice, colitis mice, colitis mice receiving berberine or SASP, and normal mice receiving berberine or SASP. Berberine or SASP was administrated via gavage one dose per day, starting either at day 1 and continued until day 7 with mice being sacrificed at day 8, or starting at day 8 after treatment with mice being sacrificed at day 14.

### Tracing the Lgr5 lineage cells

In the tracing experiment, *Lgr5-CreERT; Rosa-tdTomato* mice were divided into four groups: normal group, colitis group, colitis group receiving berberine, and normal group receiving berberine. GFP staining was used to determine the presence of Lgr5^+^ ISCs. To trace the daughter cells of Lgr5^+^ ISCs, tamoxifen and berberine were administrated at day 1 after given DSS, and the mice were sacrificed at day 8. Tomato^+^ cells were analyzed.

### H/E and Alcian blue staining

The rectum segments 2 cm above the anal canal were removed and put into a roll, which was fixed in 4% paraformaldehyde overnight. On the second day, the samples were dehydrated and embedded in paraffin. For the cryostat section, rectal samples were embedded in OCT and frozen in liquid nitrogen. Rectal samples were sectioned at 4 μm thickness and then prepared for staining. H/E staining and Alcian Blue staining were performed following standard protocols.

### Histological score

Histological score was evaluated following the standard protocol, which consists of two different scores: epithelium score and infiltration score. For the epithelium score, normal=0; loss of goblet cells=1; loss of goblet cells in large areas=2; loss of crypts=3; loss of crypts in large areas=4. For the infiltration score, no infiltration=0; infiltration around the crypt=1; infiltration reaching the muscularis mucosa=2; infiltration to the muscularis mucosa and thickening of mucosa with abundant edema=3; infiltration to submucosa=4.

### Immunohistochemical staining

Tissue sections were dewaxed, rehydrated, and permeabilized with 1% Triton X100 in PBS for 15 min. For antigen recovery, sections were put into 99°C citrate buffer or EDTA buffer for 30 min. The sections were blocked with 20% goat serum in PBS at room temperature for 1 h, then incubated with primary antibody (1:100) at 4°C overnight. On the second day, the sections were firstly rewarmed at 37°C for 1 h. For immunofluorescent staining, fluorescence-conjugated secondary antibody of corresponding species and DAPI were added at dilution 1:100 for 1 h at 37°C, and then sealed. The following primary antibodies were used: CD45 (abcam, ab10558), Ki67 (Invitrogen, pa5-19462), E-Cadherin (BD, 610182), β-Catenin (BD, 61053). DAB staining was performed following the standard protocol. The following antibodies were used: p-STAT3 (cell signaling, 9145), GFP (Cell Signaling, 2956), Vimentin (abcam, ab92547), CD45 (abcam, ab10558), CD31 (abcam, ab56299), EpCAM (abcam, ab221552). All antibodies were diluted 1:100.

### Primary colorectal stromal cell and HCT116 culture

Mice were euthanized and the colorectal tract was collected, washed for 10 min in PBS containing 0.01 mM DTT (Sigma) and 0.3 mM EDTA to remove the mucus, and then placed in PBS containing 0.3 mM EDTA for 10 more minutes. The tissue was then cut into 3–4 mm pieces, digested with type VIII collagenase at 37°C for 1.5 h. The digested juice was filtered through a 70-μm cell strainer. After centrifuging at 1000 rpm for 5 min, the cells were re-suspended in MEM Alpha medium containing 15% fetal bovine serum and cultured in 12-well plates at 37°C in a humidified atmosphere with 5% CO_2_. These cells were considered stromal cells.

Human colon cancer cell HCT-116 was cultured in RPMI 1640 medium containing 10% fetal bovine serum at an environment of 37°C with 5% CO_2_.

### siRNA transfection

For *in vitro* knock-down experiments, we used Lipofectamine^TM^2000 and Stealth RNAi ^TM^ negative control kit from Invitrogen. The sequences of *Arntl* siRNA were: 5′-CGAAGUCGAUGGUUCAGUUTT-3′; and 5′-AACUGAACCAUCGACUUCGTA-3′. The siRNA transfection was performed following the standard procedure provided by Invitrogen.

### Western blot

The colorectal tissues were taken out from −80°C and placed on ice. RIPA buffer containing protein inhibitors was added in 1:10 ratio according to the weight of the tissues. After grinding for 2–3 cycles (65HZ, 90s), the supernatant was collected and the protein concentration was determined using Pierce^TM^ BCA Protein Assay Kit (Pierce, USA). Protein samples were electrophoresed with 10% SDS-PAGE and transferred to PVDF membrane (Millipore), blocked with 5% non-fat milk for 1 h, incubated with primary antibodies at 4°C overnight, followed by incubation with secondary antibodies. The blots were visualized and quantified with the FluoChem M system (ProteinSimple). The following antibodies were used: β-Catenin (BD, 61053), p-Stat3 (Cell Signaling, 9145), Stat3 (Cell Signaling, 9139), p-ERK (Cell Signaling, 9106), ERK (Cell Signaling, 9102), p-Akt (Cell Signaling, 4060), Akt (Cell Signaling, 9272), β-Actin (Santa Cruz, 47778), Arntl (Cell Signaling, 14020), Clock (Cell Signaling, 5157), IL-6 (abcam, 229381), TNFα (Cell Signaling, 11948), Wnt6 (abcam, 154144), Wnt10a (abcam, 106522), PMCA (Santa Cruz, 271917). All antibodies were diluted 1:1000.

### Quantitative PCR

Total RNA was isolated from tissues or cells with Trizol regent (Invitrogen), and 1 μg RNA was reversed transcribed into cDNA with the cDNA Synthesis Kit (Thermo), which was used for quantitative PCR analysis (Roche LightCycler480II). All the primers were based on previously published studies and purchased from Shanghai Sangon Biotech Co., Ltd. The sequences of the primers were listed in Table S1.

### RNA sequencing and analysis

Total RNA was extracted from the fresh rectal samples of normal mice, colitis mice, normal mice receiving berberine, and colitis mice receiving berberine. Ribo-Zero Gold rRNA removal kit H/M/R (Illumina) was used for rRNA depletion. Total RNA sample QC was performed on 2100 Bioanalyzer (Agilent) using Agilent RNA 6000 Nano Kit. Libraries were constructed following standard steps, with library being single-strand circle DNA (ssCir DNA). For the purpose to make DNA nanoball (DNB), the library was amplified with phi29 (Thermo Fisher Scientific). For sequencing, DNBs were transformed to single end 50 bases reads and sequence on the BGISEQ-500 platform (BGI-Shenzhen, China). Filtering reads and generating FASTQ format were achieved by SOAPnuke (v1.5.2). Bowtie2 (v2.2.5) was used to align clean reads to the reference mouse genome with parameters and RSEM (v1.2.12) was used to calculate gene expression. In this study, different expression genes (DEGs) that met statistical significance (*P*-value ≤0.05) and expression fold changes (fold change ≥0) were analyzed further. In pathway analysis and GO analysis, all candidate DEGs were classified according to official classification with the KEGG or GO annotation result and hyper (a function of R) was performed in GO and pathway functional enrichment. In GO analysis, stage-specific gene signature was analyzed in Biological Process. The *P*-value calculating formula was performed on wiki (https://en.wikipedia.org/wiki/Hypergeometric_distribution). False discovery rate (FDR) was calculated for each *p* value with FDR ≤ 0.01 and was defined as significantly enriched.

### Flow cytometry and cell sorting

The fresh rectal samples were digested with collagenase VIII following the standard protocol. Released cells were passed through a 70-μm strainer and cells were collected after being centrifuged at 1000 rpm for 5 min. For rectal lymphocyte isolation, at the interface of a 40:80% Percoll (GE Healthcare) gradient, lamina propria lymphocytes could be collected after being centrifuged at 2500 rpm for 20 min. For rectal lymphocytes analysis, lamina propria lymphocytes were stained with Fixable Viability dye (BD Bioscience) for 20 min. After that, the isolated cells were stained with lymphocyte surface markers for 30 min. The surfaces markers were as follows: APC-F4/80, APC/Cyanine7-CD4, APC/Cyanine7-Gr1, FITC-CD3, PerCP/Cyanine5.5-CD11b, APC-CD86, and BV510-CD8a (all from BioLegend), FITC-MHCII (eBioscience), and Alexa Fluor 700-CD45.2 (BD). All data collected were re-analyzed using FlowJo (Tree Star). For cell sorting, the cells were stained with the following antibodies for 30 min: FITC-CD31, PE-CD45, PE-CD51, and PE-CD326 (also known as EpCAM) (all from BioLegend). Cell sorting was performed on S3e^TM^ cell sorter (Bio-Rad). For primary colon stromal marker analysis, cells were stained with the following markers for 30 min: PE/Cyanine7-Epcam, FITC-CD31, PE/Dazzle-CD45, FITC-CD29, PE-CD51 (all from BioLegend), and Vimentin (abcam, ab92547). In addition, Vimentin required secondary antibodies.

### Statistical analysis

All data were presented as mean ± SEM and all experiments were repeated at least three times. Statistical analysis and plotting were processed with GraphPad Prism 8. Unpaired two-tailed Student’s *t* test was used to determine the significance of the differences between two groups. *p*<0.05 were considered significant.

## Supplementary Information

**Additional file 1: Table S1.** Primer sequences used in the study. **Fig. S1.** Determining the optimal dose of BBR or SASP on colitis treatment. **Fig. S2.** Comparison of BBR and SASP on colitis after DSS withdrawal. **Fig. S3.** Gene expression profiling of rectal samples of BBR-treated normal mice. **Fig. S4.** BBR promotes the expression of mineral absorption genes. **Fig. S5.** Comparison of phenotypes at 03, 09, 15, and 21 of the day in DSS-induced colitis mice. **Fig. S6.** Gene expression profiling of samples of SASP-treated colitis mice. **Fig. S7.** Isotype controls for immune cell flow cytometry analysis. **Fig. S8.** Genotyping of the *Lgr5-CreERT; Rosa-tdTomato* mice. **Fig. S9.** The effect of BBR on β-Catenin and other signaling molecules in HCT116 cells. **Fig. S10.** Verification of the colonic stromal cells. **Fig. S11.** The effect of BBR on circadian gene expression in colorectal immune and epithelial cells.

**Additional file 2.** Original gels and blots.

## Data Availability

The mouse RNA-seq data used in this study were uploaded onto the GEO database (NCBI) under the accession Series GSE197342 [75] and GSE216260 [76], which are publicly available. All other data generated or analyzed during this study are included in this published article and its additional files.

## Abbreviations

BBR: Berberine
SASP: Sulfasalazine
IBD: Inflammatory bowel disease
ISC: Intestinal stem cell
