# Supplementary material for: Berberine increases stromal production of Wnt molecules and activates Lgr5+ stem cells to promote epithelial restitution in experimental colitis
Source: BMC Biol. 2022 Dec 17;20:287. doi: 10.1186/s12915-022-01492-z (PMC9759859; doi:10.1186/s12915-022-01492-z)
Supplement: Supplementary file 1 — Additional file 1: Table S1. Primer sequences used in the study. Fig. S1. Determining the optimal dose of BBR or SASP on colitis treatment. Fig. S2. Comparison of BBR and SASP on colitis after DSS withdrawal. Fig. S3. Gene expression profiling of rectal samples of BBR-treated normal mice. Fig. S4. BBR promotes the expression of mineral absorption genes. Fig. S5. Comparison of phenotypes at 03, 09, 15, and 21 of the day in DSS-induced colitis mice. Fig. S6. Gene expression profiling of samples of SASP-treated colitis mice. Fig. S7. Isotype controls for immune cell flow cytometry analysis. Fig. S8. Genotyping of the Lgr5-CreERT; Rosa-tdTomato mice. Fig. S9. The effect of BBR on β-Catenin and other signaling molecules in HCT116 cells. Fig. S10. Verification of the colonic stromal cells. Fig. S11. The effect of BBR on circadian gene expression in colorectal immune and epithelial cells. [file 12915_2022_1492_MOESM1_ESM.docx]

**Table S1. Primer sequences used in the study.**

| Gene(mouse) | Forward Primer | Reverse Primer |
| --- | --- | --- |
| IL6 | GTTGCCTTCTTGGGACTGATG | GACTCTGGCTTTGTCTTTCTTGTT; |
| IL1b | GCAACTGTTCCTGAACTCAACT | ATCTTTTGGGGTCCGTCAACT |
| TNF-α | CCCTCACACTCAGATCATCTTCT | GCTACGACGTGGGCTACAG |
| IFN-γ | CTGCTGATGGGAGGAGATGTCT | TGCTGTCTGGCCTGCTGTTA |
| Ptgs2 | CAGATGACTGCCCAACTCCC | TGAACCCAGGTCCTCGCTTA |
| Arntl | GCAGTGCCACTGACTACCAAGA | TCCTGGACATTGCATTGCAC |
| Clock | CCTATCCTACCTTGGCCACACA | TCCCGTGGAACCTAGAC |
| Cry1 | CCCAGGCTTTTCAAGGAATGGAACA | TCTCATCATGGTCATCAGACAGAGG |
| Cry2 | GGGACTCTGTCTATTGGCATCTG | GTCACTCTAGCCCGCTTGGT |
| Per1 | CAGGCTAACCAGGAAATCTACCAGC | CACAGCCACAGAGAAGGTGTCCTGG |
| Per2 | GGCTTCACCATGCCTGTTGT | GGAGTTATTTCGGAGGCAAGTGT |
| Per3 | CTGCTCCAACTCAGCTTCCTTT | TTAGACAGCAAGGCTCTGGTTCT |
| Npas2 | GTATGCACAGAGCCAAGTGATGTT | TGCTCACTGTGCAGAGACGTTG |
| Nr1d1 | TTTTTCGCCGGAGCATCCAA | ATGTCGGCAAGCATCCGTTG |
| B2M | GCTATCCAGAAAACCCCTCAAATTCA | GCAGGCGTATCTATCAGCCTCAGTG |
| S100g | CCTGCAGAAATGAAGAGCATTTT | CTCCATCGCCATTCTTATCCA |
| Slc5a1 | TTGGAGTCCTCTGGGATGTC | GCCATCATCCTCTTCGTCAT |
| Slc46a1 | ATGTTAGCTCTAACCACAGTCG | AACAAAATGCTGCGTATAGAGC |
| Slc26a3 | GGTTGGGAACATGAGTCTTGGA | GCCGAAGCTGTCTCCTATGG |
| Slc34a2 | CTATTCCGCCCTGGTTCTC | GAAAATGCAGAGCGTCTTCC |
| VDR | AGGCCCACACTCAGCTTCT | ACAGGTCCAGGGTCACAGAG |
| Atp2b1 | GCACCAAGTTGAAAACATCTCCC | TCTCCACAAAGTGCATTATCCCC |
| Atp2b4 | GGTCAGGTCATCTCTGCAATAC | CATCTCGGCAAGGTCAATCT |

**Fig. S1**

**
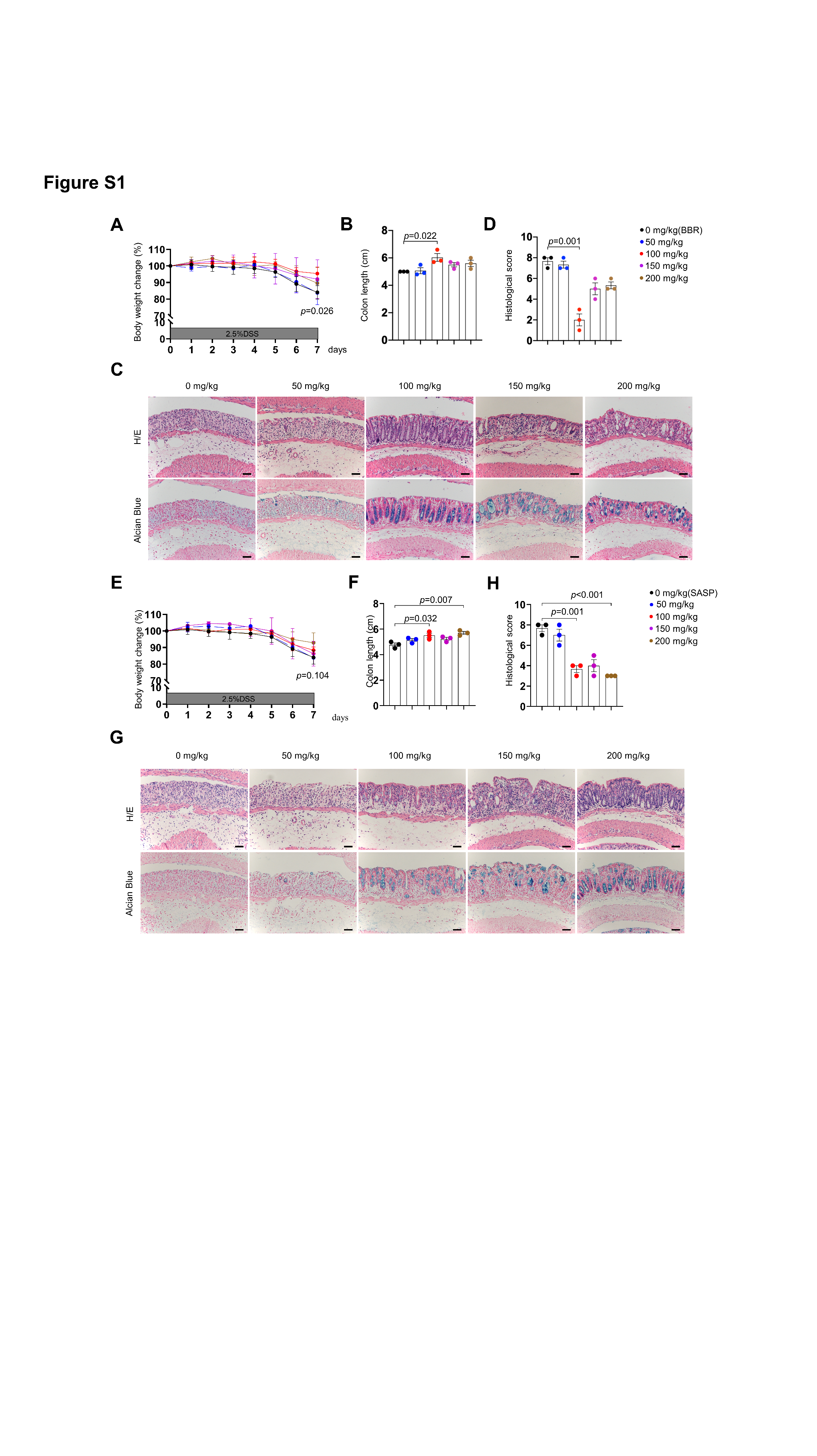
**

**Fig. S1 Determining the optimal dose of BBR or SASP on colitis treatment.A**-**D** Colorectal samples were harvested after 7 days of DSS treatment in adult male mice, which were treated with different doses of BBR starting from day 1. We determined the body weights (**A**), colon length (**B**), and histological scores (**D**) and performed H/E and Alcian Blue staining of rectal sections (**C**). **E**-**H** Colorectal samples were harvested after 7 days of DSS treatment in adult male mice, which were treated with different doses of SASP starting from day 1. We determined body weights (**E**), colon length (**F**), and histological scores (**H**) and performed H/E and Alcian Blue staining of rectal sections (**G**). *n*=3 per group. Scale bars, 50 μm. Data are presented as means±SEM. Unpaired two-tailed Student’s *t* test was applied. *p*<0.05 was considered as statistically significant.

**Fig. S2**

**
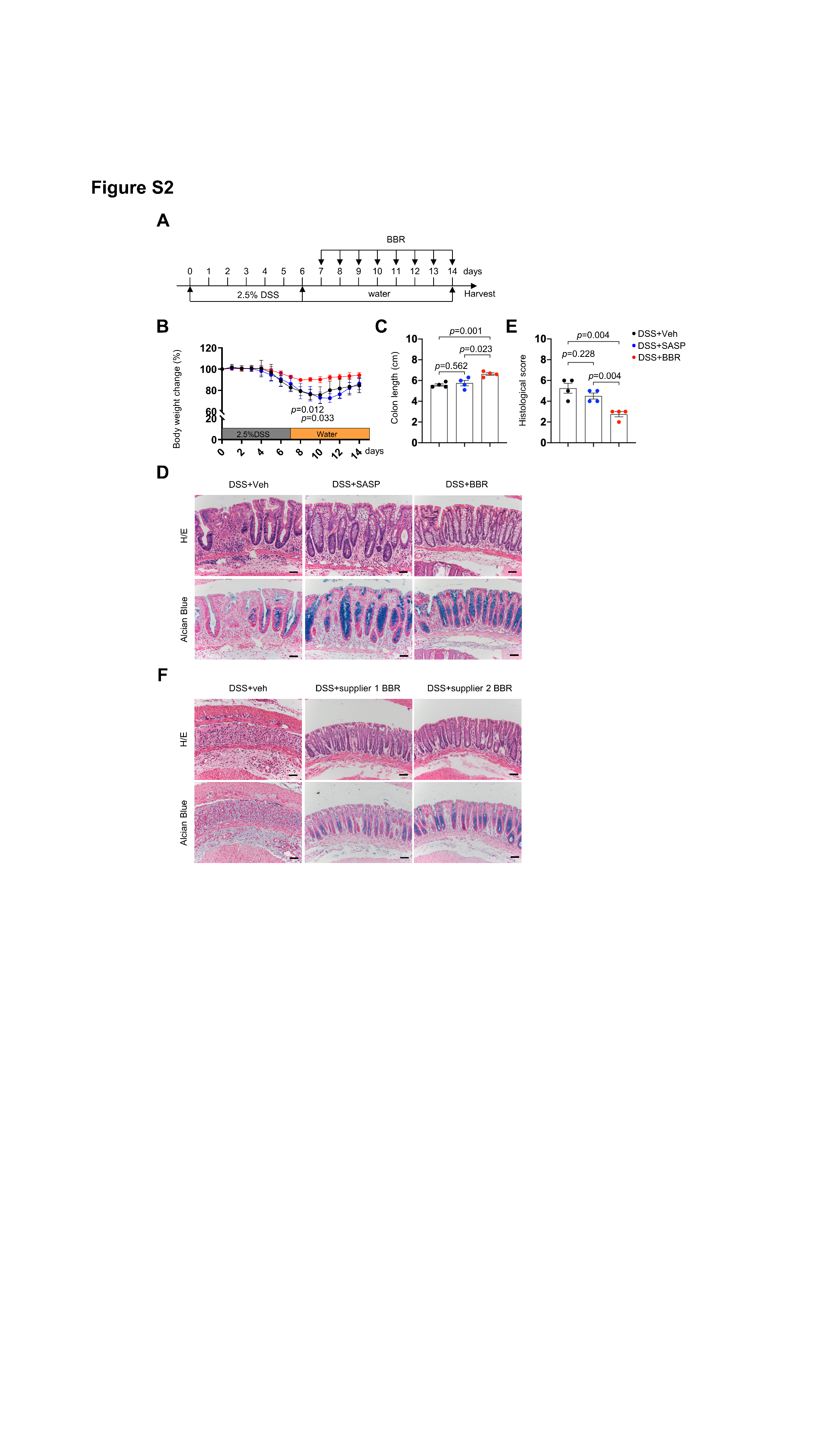
**

**Fig. S2 Comparison of BBR and SASP on colitis after DSS withdrawal.** **A** Diagram showing the times of DSS and BBR treatment and colorectal sample collection. The mice were treated with DSS for 7 days and then on DSS-free water for 7 more days. BBR or SASP was administrated at the beginning of DSS withdrawal. **B-E** We determined body weights (**B**), colon length (**C**), and histological scores (**E**) and performed H/E and Alcian Blue staining of rectal sections (**D**). *n*=3 per group. Scale bars, 50 μm. **F** BBR from two different suppliers were compared. *n*=3 per group. Scale bars, 50 μm. Data are presented as means±SEM in (**B, C** and **E**). Unpaired two-tailed Student’s *t* test was applied in (**B, C** and **E**). *p*<0.05 was considered as statistically significant.

**Fig. S3**

**
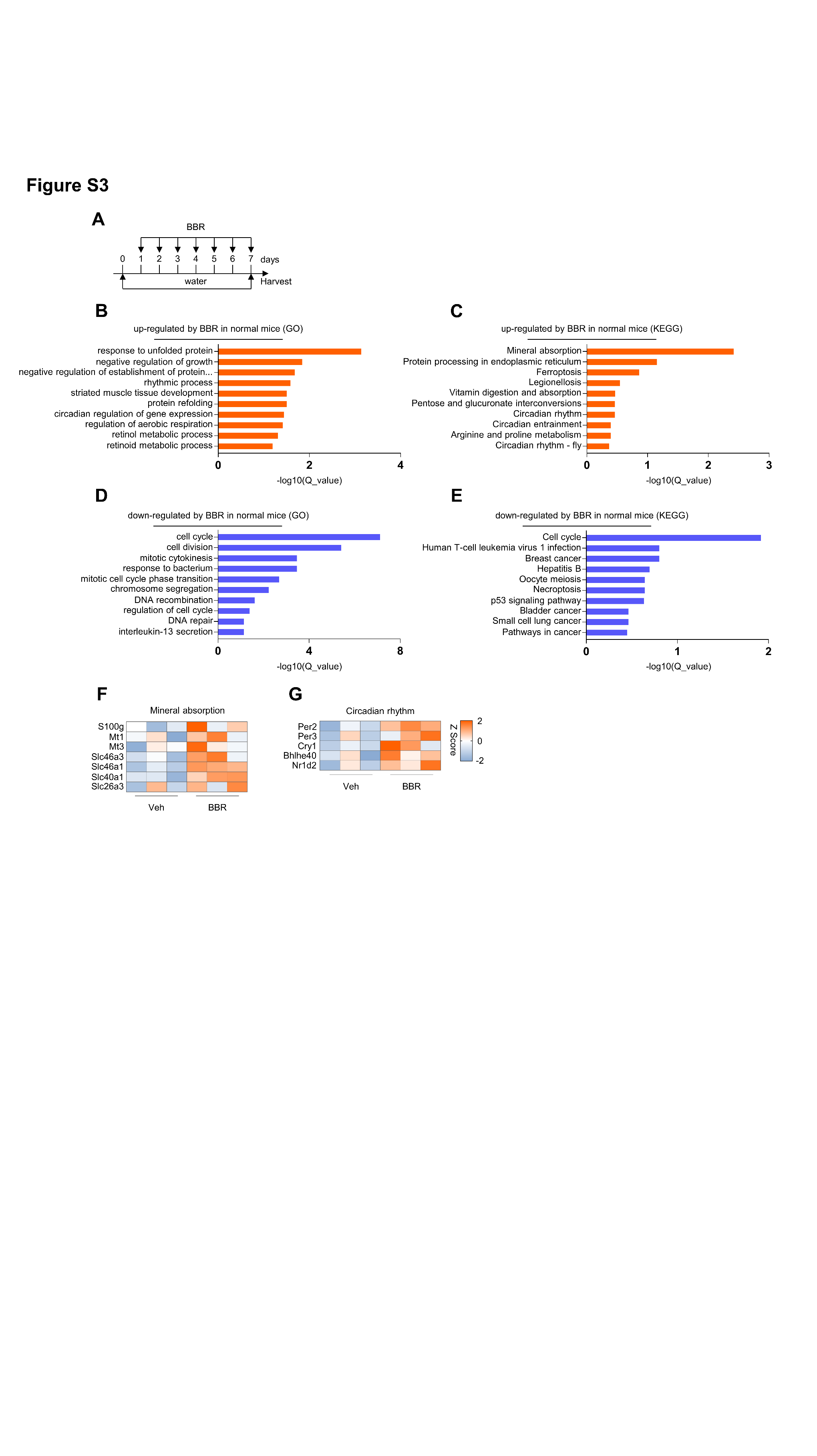
**

**Fig. S3 Gene expression profiling of rectal samples of BBR-treated normal mice. A** Diagram showing the times of BBR treatment and sample collection. **B, C** GO and KEGG pathway analyses showing up-regulated gene clusters by BBR in normal mice. **D, E** GO and KEGG pathway analyses showing down-regulated gene clusters by BBR in normal mice. **F, G** Heatmaps of mineral absorption and circadian rhythm genes, *n*=3 per group.

**
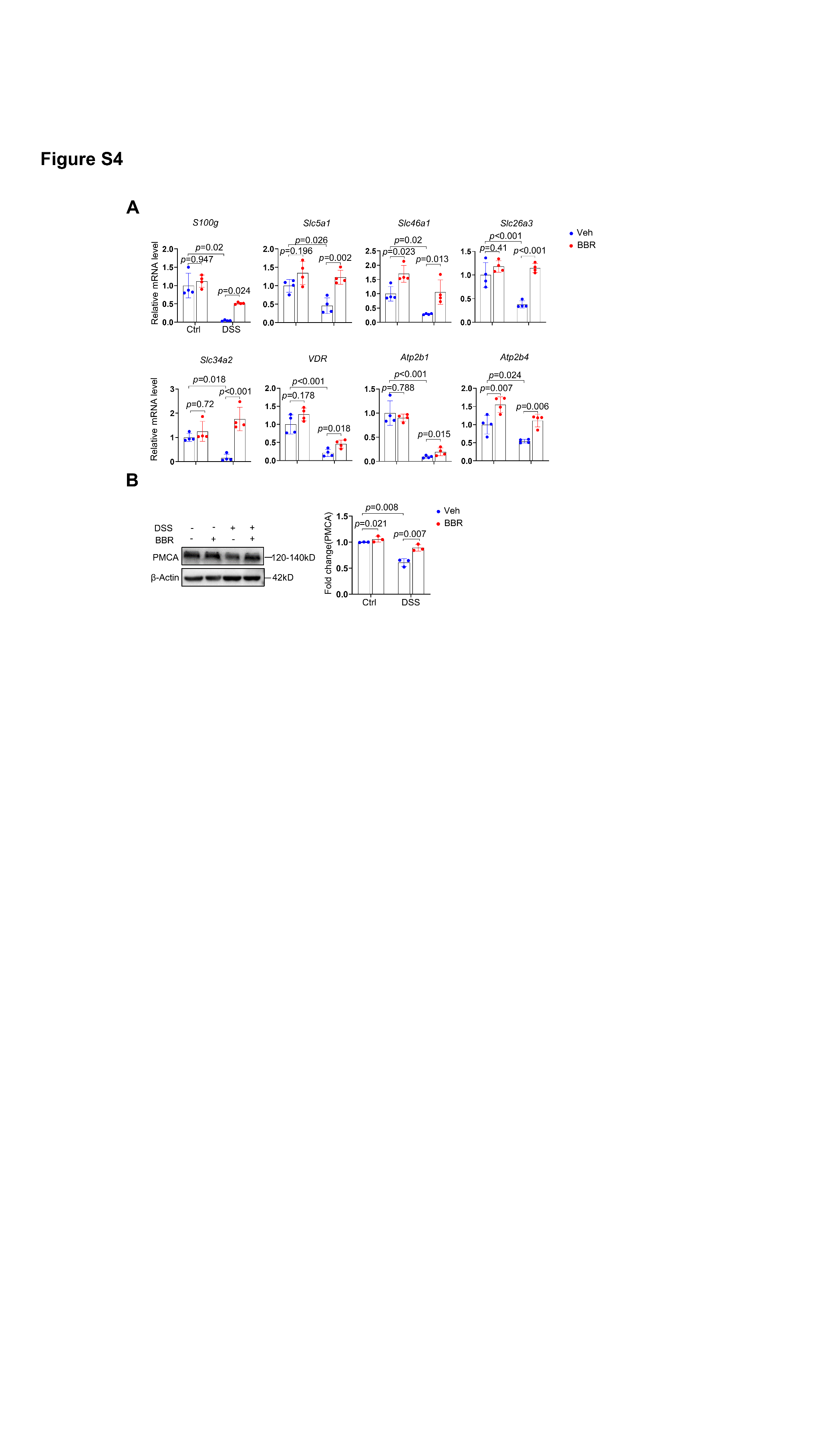
Fig. S4**

**Fig. S4 BBR promotes the expression of mineral absorption genes.** **A** qPCR analysis of various mineral absorption-related genes in colon samples of normal mice, colitis mice, normal mice receiving BBR, and colitis mice receiving BBR, n=4 per group. **B** Western blot analysis revealed that the colon samples of colitis mice showed a decrease in PMCA proteins, which was rescued by BBR. Right panels: quantitation data. n=3. Data are presented as means±SEM in (**A, B**). Two-way ANOVA with Fisher’s LSD post hoc analysis (α = 0.05) was applied in (**A, B**). p<0.05 was considered as statistically significant.

**Fig. S5**

**
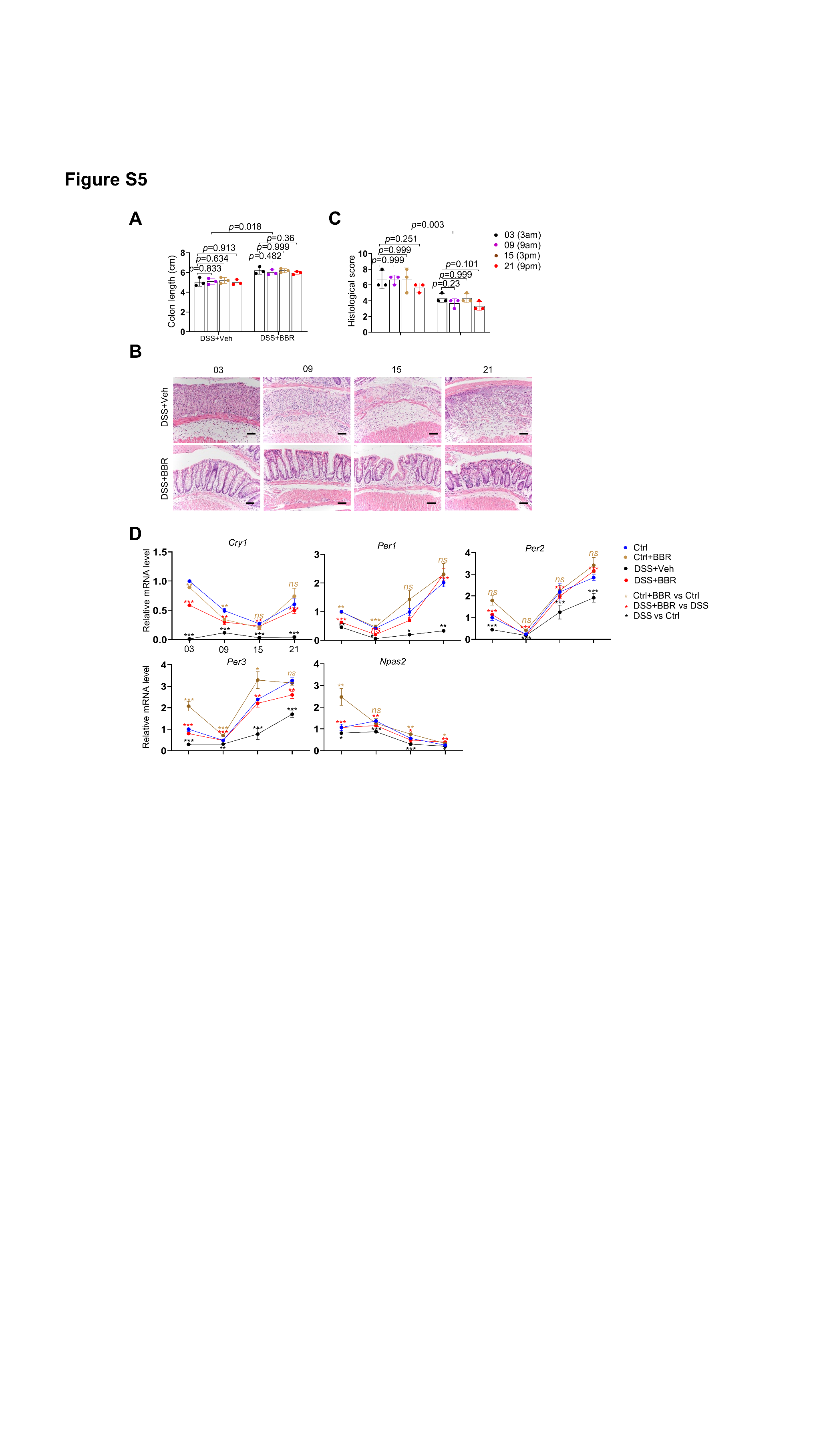
**

**Fig. S5 Comparison of phenotypes at 03, 09, 15, and 21 of the day in DSS-induced colitis mice. A-C** Colorectal samples were harvested at different time of the day after 7 days of DSS treatment in adult male mice, which were treated with BBR starting from day 1. We determined colon length (**A**) and histological scores (**B**) and performed H/E and Alcian Blue staining of rectal sections (**C**). **D** qPCR analysis of circadian rhythm genes in rectal samples of normal mice, colitis mice, normal mice receiving BBR, and colitis mice receiving BBR, which were collected at different time points, *n*=3 per group. Data are presented as means ± SEM. Unpaired two-tailed Student’s *t* test was applied in (**A**). Two-way ANOVA with Fisher’s LSD post hoc analysis (α = 0.05) was applied in (**D).** *p*<0.05 was considered as statistically significant. * *p*<0.05, ** *p*<0.01, *** *p*<0.001.

**Fig. S6**

**
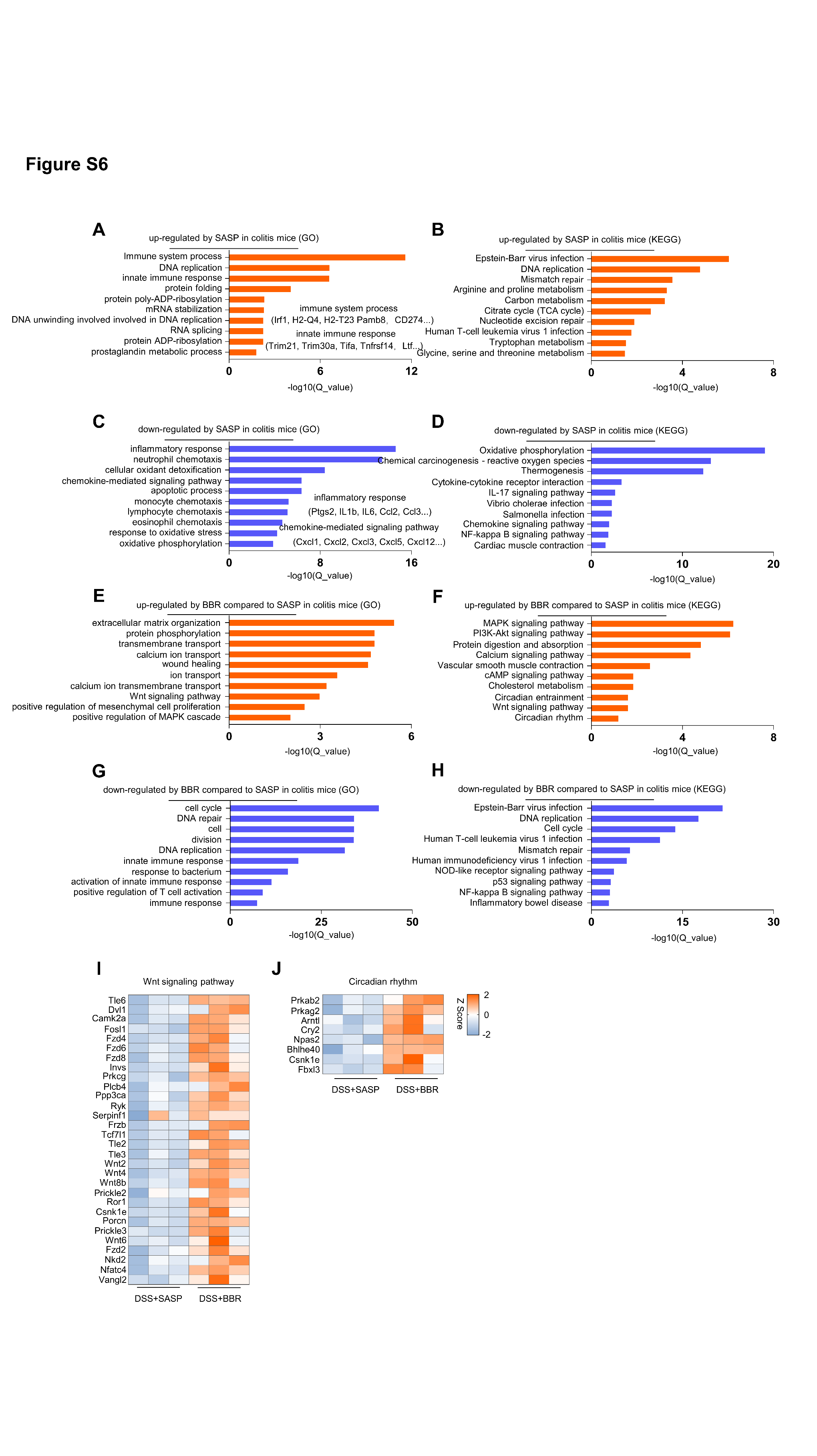
**

**Fig. S6 Gene expression profiling of samples of SASP-treated colitis mice. A, B** GO and KEGG pathway analyses showing up-regulated gene clusters by SASP in colitis mice. **C, D** GO and KEGG pathway analyses showing down-regulated gene clusters by SASP in colitis mice. **E, F** GO and KEGG pathway analyses showing up-regulated gene clusters by BBR in colitis mice compared to SASP-treated colitis mice. **G, H** GO and KEGG pathway analyses showing down-regulated gene clusters by BBR in colitis mice compared to SASP-treated colitis mice. *n*=3 per group. **I, J** heatmaps of Wnt signaling pathways genes (**I**) and circadian rhythm genes (**J**) up-regulated by BBR compared to SASP-treated colitis samples. *n*=3 per group.

**Fig. S7**

**
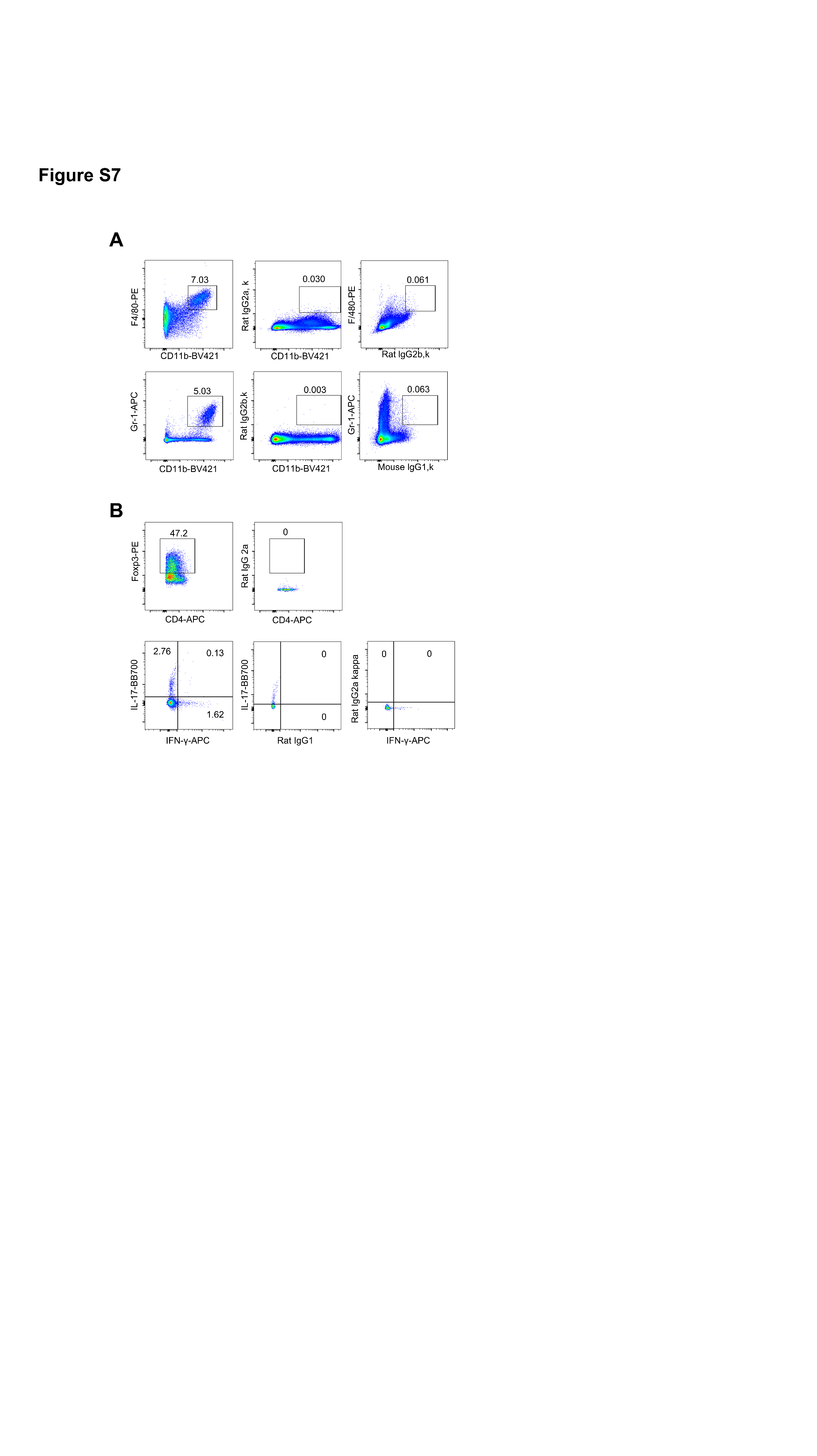
**

**Fig. S7 Isotype controls for immune cell flow cytometry analysis.** Representative FACS plots showing gating strategy for granulocytes and macrophages (**A**) and T cells (**B**).

**Fig. S8**

**
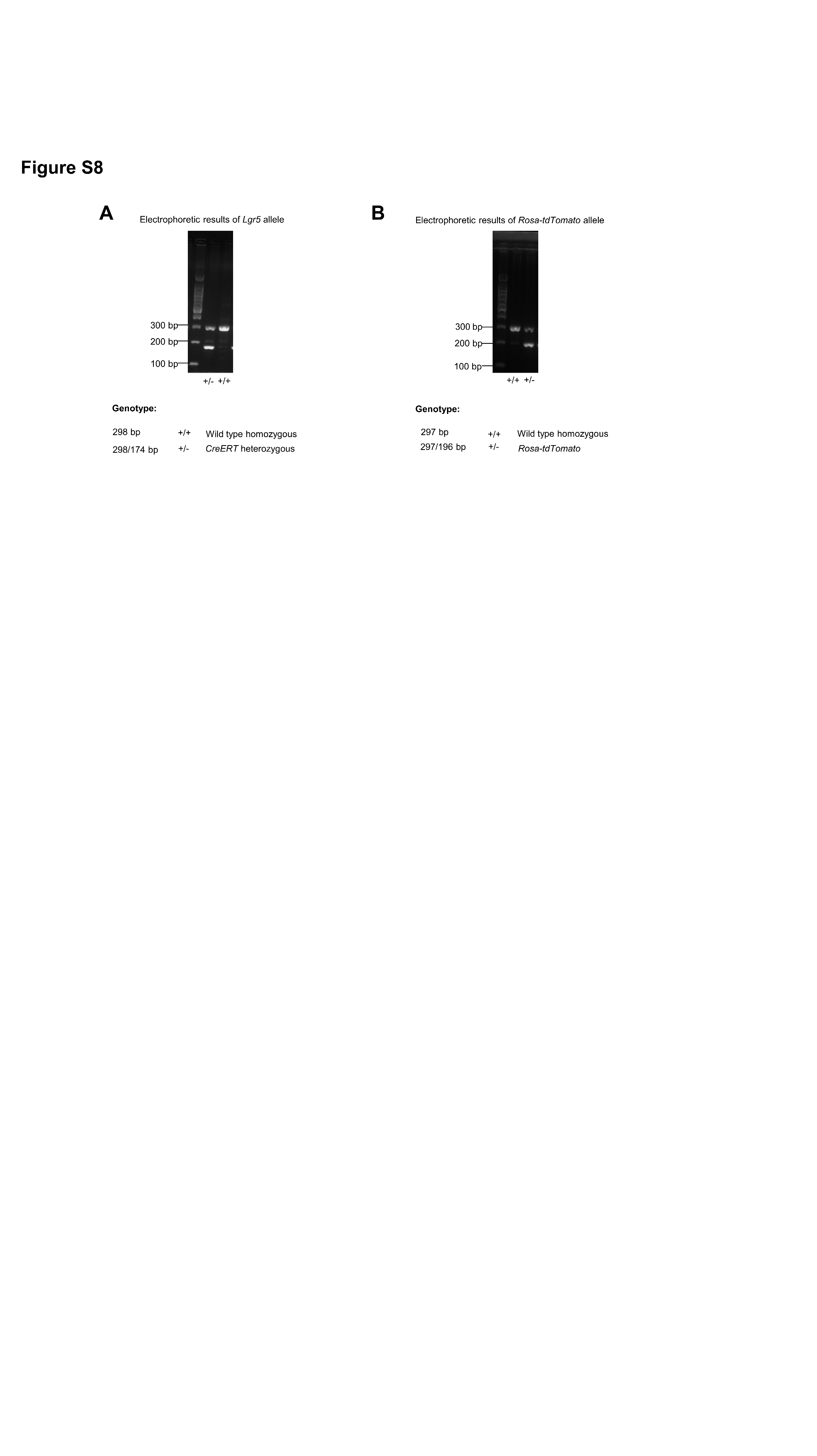
**

**Fig. S8 Genotyping of the *Lgr5-CreERT; Rosa-tdTomato* mice.** Representative PCR results showing the deletion of one allele of *Lgr5* (**A**) and the floxed STOP codon in one allele of Tomato (**B**) in *Lgr5-CreERT; Rosa-tdTomato* mice.


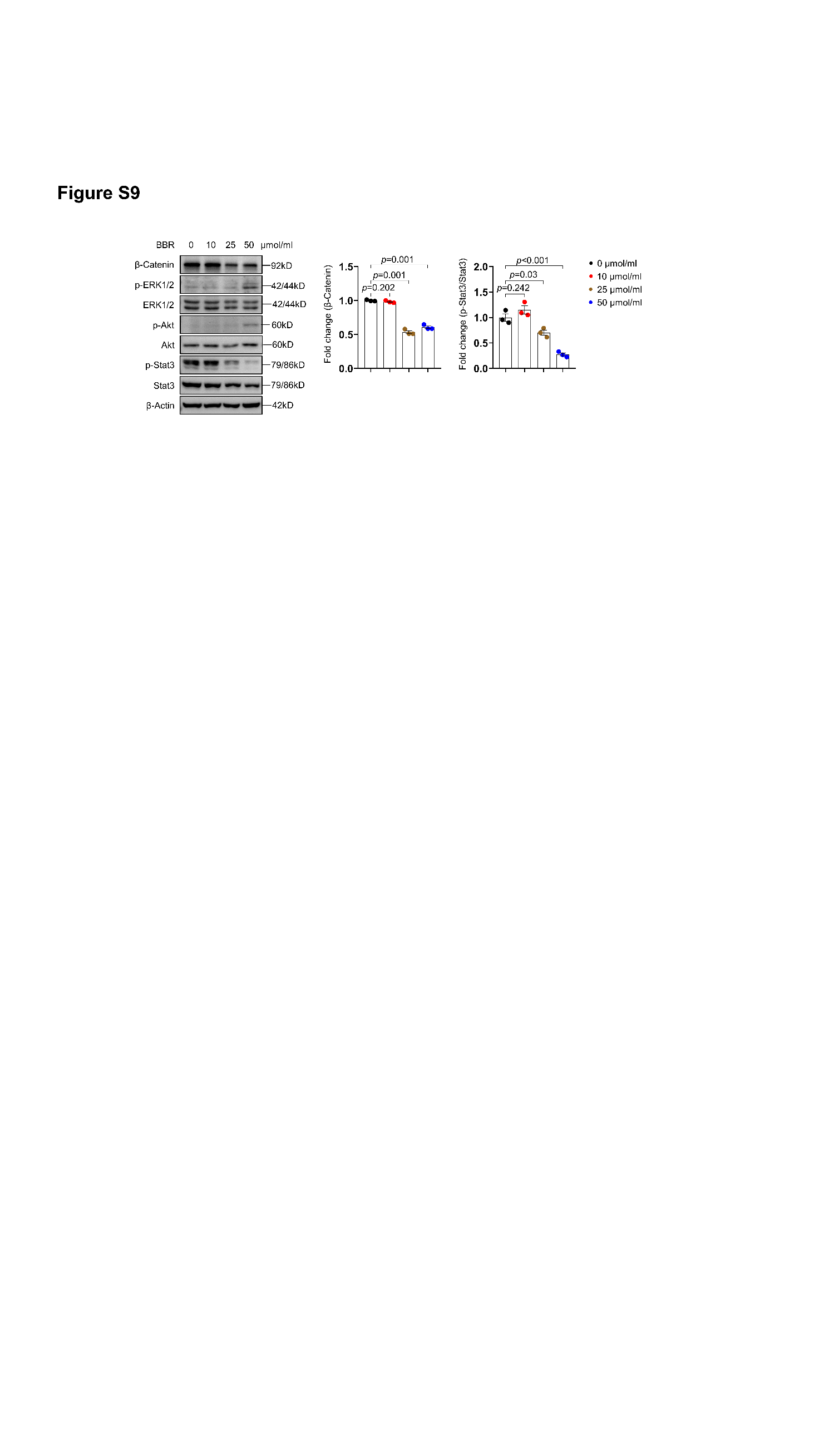
**Fig. S9**

**Fig. S9 The effect of BBR on β-Catenin and other signaling molecules in HCT116 cells.** WB results showed activation of β-Catenin and other signaling molecules in epithelial cell line HCT116 treated by different dose of BBR after 24 hrs of treatment, *n*=3 per group. Right panels: quantitation data. Data are presented as means±SEM. Unpaired two-tailed Student’s *t* test was applied. *p*<0.05 was considered as statistically significant.

**Fig. S10**

**
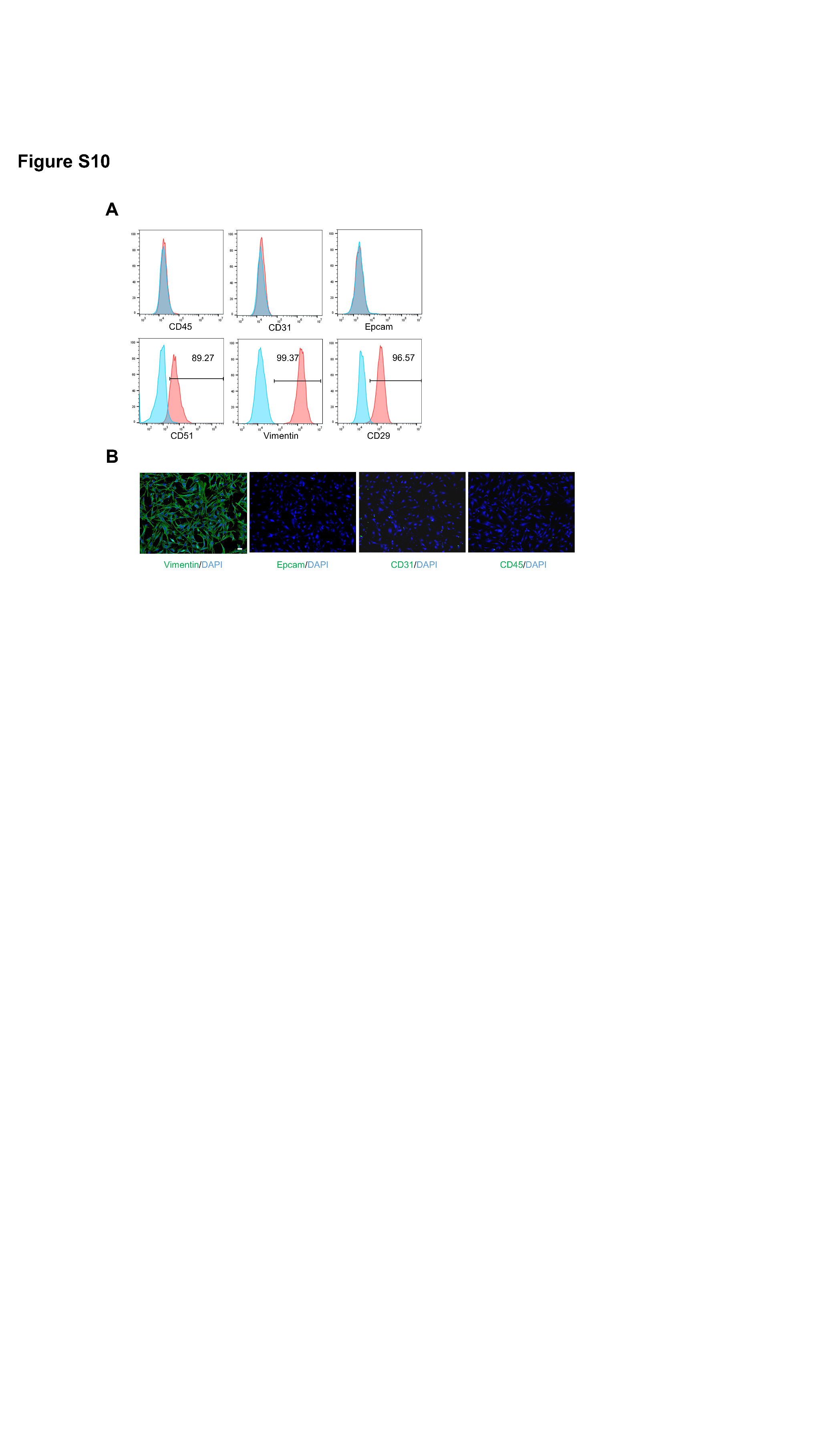
**

**Fig. S10 Verification of the colonic stromal cells. A** Stromal cells were isolated based on the protocol described in Fig. 5F. The representative flow cytometry plots were presented for CD31, CD45, CD51, EpCAM, Vimentin, and CD29. **B** Immunostaining of the sorted cells for Vimentin, EpCAM, CD31, and CD45.

**Fig. S11**

**
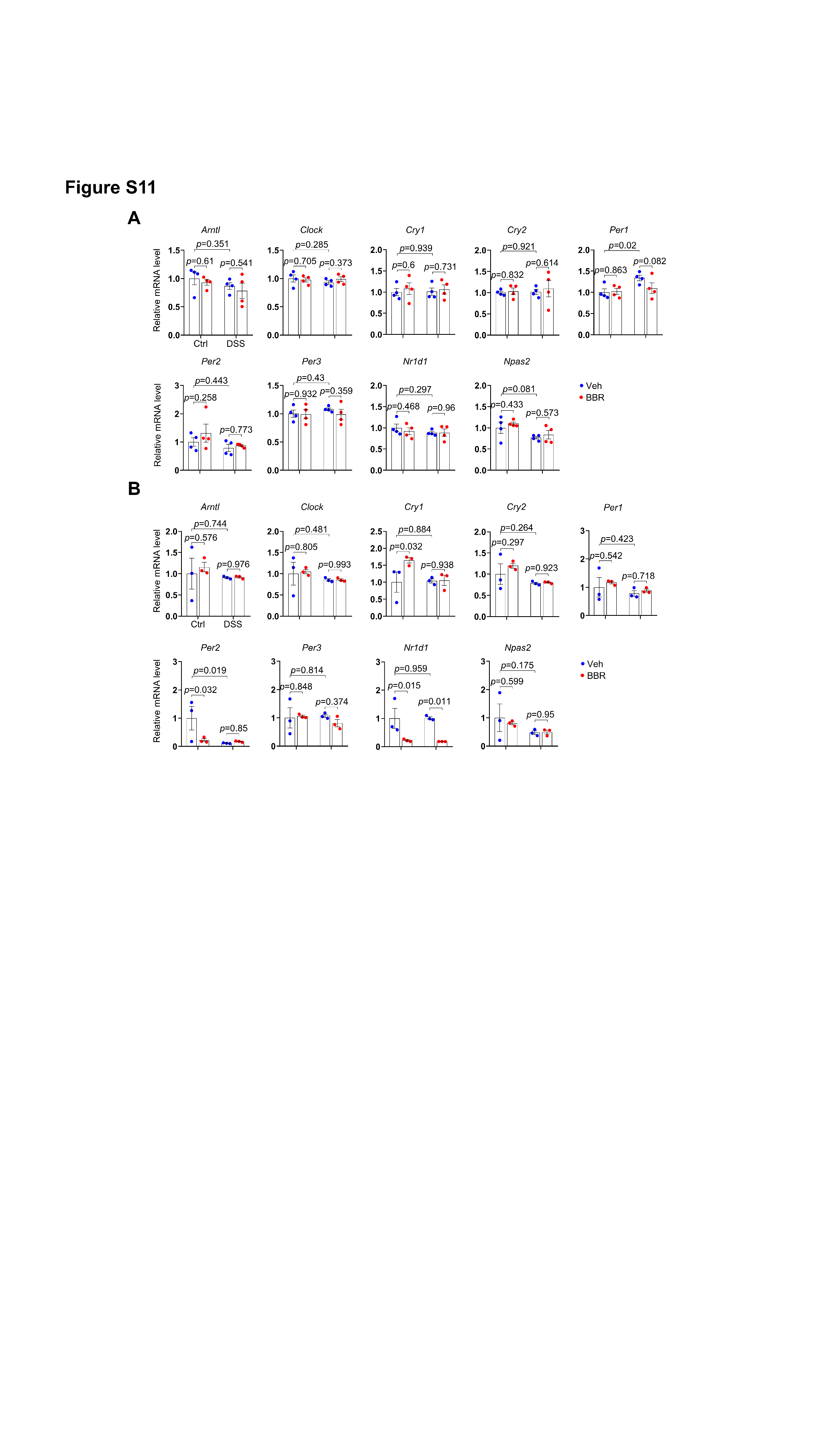
**

**Fig. S11 The effect of BBR on circadian gene expression in colorectal immune and epithelial cells. A** qPCR analysis of *Arntl*, *Clock*, *Cry1*, *Cry2*, *Per1*, *Per2*, *Per3*, *Nr1d1* and *Npas2* in immune cells. **B** qPCR analysis of *Arntl*, *Clock*, *Cry1*, *Cry2*, *Per1*, *Per2*, *Per3*, *Nr1d1* and *Npas2* in epithelial cells, *n*=3 per group. Data are presented as means±SEM in (**A, B**). Two-way ANOVA with Fisher’s LSD post hoc analysis (α = 0.05) was applied in (**A, B**). *p*<0.05 was considered as statistically significant.
