# Supplementary material for: Berberine increases stromal production of Wnt molecules and activates Lgr5+ stem cells to promote epithelial restitution in experimental colitis
Source: BMC Biol. 2022 Dec 17;20:287. doi: 10.1186/s12915-022-01492-z (PMC9759859; doi:10.1186/s12915-022-01492-z)

Fig. 4C

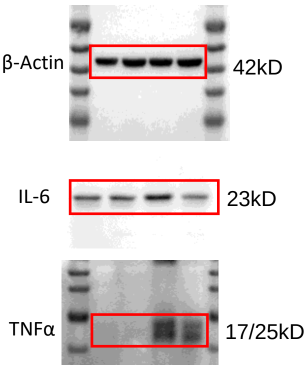

Fig. 5D

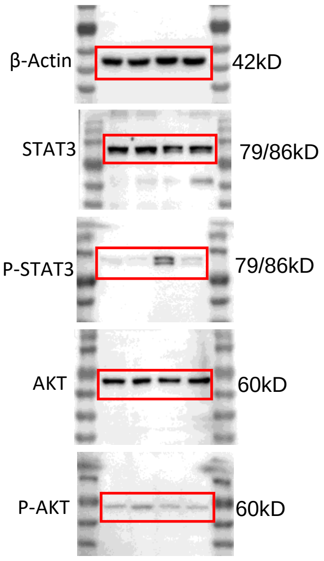

Fig. 5D

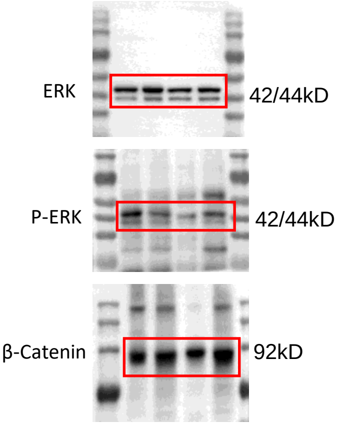

Fig. 5H

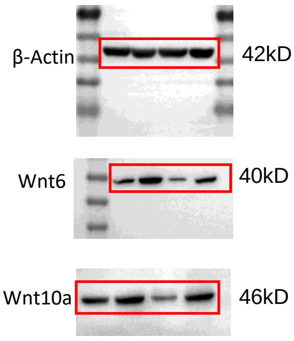

Fig. 6A

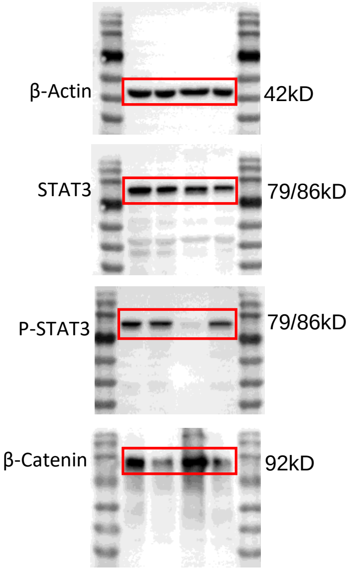

Fig. 7B

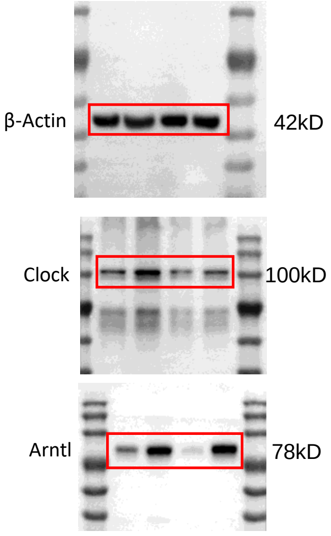

Fig. S4B

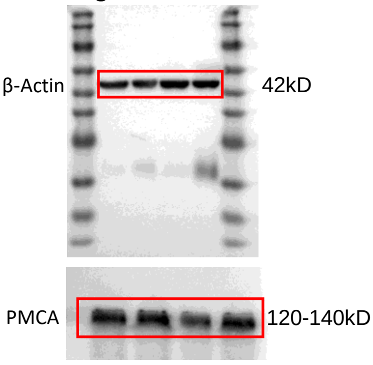

Fig. S8 Electrophoretic results of *Lgr5* allele

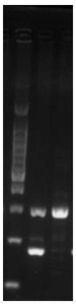

Fig. S8 Electrophoretic results of *Rosa-tdTomato* allele

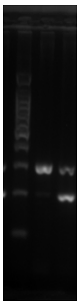

Fig. S9

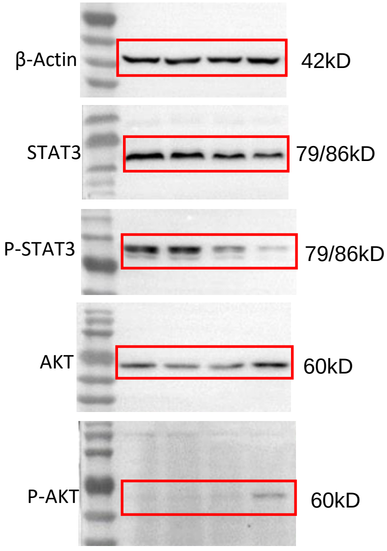

Fig. S9

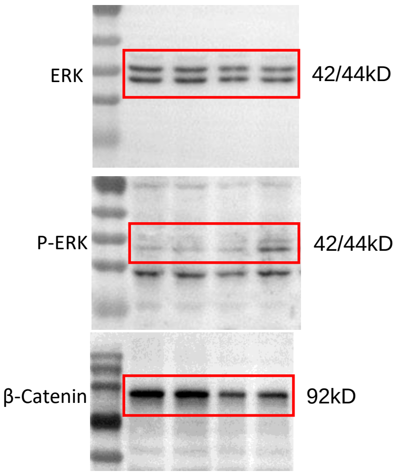

Supplement: Supplementary file 2 — Additional file 2. Original gels and blots. [file 12915_2022_1492_MOESM2_ESM.pdf]
